# Glioma-induced DNMT3A reduction in microglia promotes an anti-tumoral phenotype

**DOI:** 10.1038/s41418-026-01712-x

**Published:** 2026-03-18

**Authors:** Mathilde Cheray, Mercedes Posada-Pérez, Adamantia Fragkopoulou, Carlos F. D. Rodrigues, Adriana-Natalia Murgoci, Ahmed M. Osman, Guillermo Vázquez-Cabrera, Martin Škandík, Christine C. Hong, Pinelopi Engskog-Vlachos, Shigeaki Kanatani, Yue Li, Stefan Spulber, Lara Friess, Theodora Sylaidi, Marie-Kim St-Pierre, Lena-Maria Carlson, Anastasios Damdimopoulos, Per Uhlén, Fredrik Kamme, Klas Blomgren, Bertrand Joseph

**Affiliations:** 1 Toxicology Unit, Institute of Environmental Medicine, Karolinska Institutet, Stockholm, Sweden; 2 Division of Pediatric Oncology and Pediatric Surgery, Department of Women’s and Children’s Health, Karolinska Institutet, Stockholm, Sweden; 3 Pediatric Oncology, Astrid Lindgren Children’s Hospital, Karolinska University Hospital, Region Stockholm, Stockholm, Sweden; 4 Ionis Pharmaceuticals, Inc., Carlsbad, CA USA; 5 Department of Medical Biochemistry and Biophysics, Karolinska Institutet, Stockholm, Sweden; 6 Department of Neuroscience, Karolinska Institutet, Stockholm, Sweden; 7 Department of Medicine, Karolinska Institutet, Huddinge, Sweden; 8 Center for Neuromusculoskeletal Restorative Medicine, Hong Kong Science Park, Shatin, New Territories, Hong Kong

**Keywords:** CNS cancer, Microglia

## Abstract

Glioblastoma, IDH1 wildtype, aggressive primary brain tumors with a dismal prognosis, promote the recruitment of microglia, brain resident innate immune cells, and ultimately their activation toward a tumor-supportive phenotype that increases gliomal proliferation and invasion capability. Here, we report that upon stimulation by glioma cells, microglia transit via a reactive state holding anti-tumoral properties coupled to reduced DNA methyltransferase 3 A (DNMT3A) chromatin occupancy and DNA demethylation that promote the expression of gene sets related to the transforming growth factor beta (TGF-β)-dependent microglial homeostasis and the microglial sensome. We find that upon repression of *Dnmt3a* expression in microglia, those cells maintain anti-tumoral attributes in vitro and in vivo. In a syngeneic immunocompetent glioblastoma mouse model, brain delivery of antisense oligonucleotide targeting *Dnmt3a* expression led to microglial activation and reduced tumor growth. Taken together, our results reveal the involvement of DNA demethylation in the control of glioma cells-induced microglia activation and indicate that microglial DNMT3A is a potentially therapeutic target to treat brain neoplasms such as glioblastoma that include a microglial component.

**Figure:**
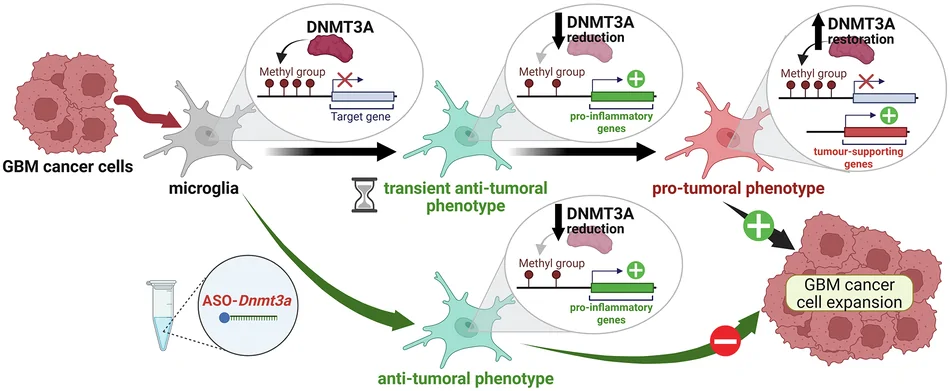

## Introduction

Glioblastoma, IDH-wildtype (GB), is a highly aggressive primary brain tumor with limited therapeutic options and a dismal prognosis for patients [1]. The intrinsic capacity of these high-grade gliomas to infiltrate the surrounding brain tissue impedes surgical resection and contributes significantly to the failure of current therapeutic treatments, which predictably results in high rates of early recurrence. Despite multimodal therapy with the concomitant use of radiation and adjuvant treatment (e.g., temozolomide, bevacizumab), the overall median survival for patients with GB is still restricted to about 15 months [1]. Thus, primary high-grade gliomas, such as GB, with their significant mortality and morbidity, remain one of the major challenges of today’s oncology.

One emerging view is that if new therapeutic strategies are to be found for this devastating neoplasm, one will have to take into account that GB tumors are heterogeneous with respect to the composition of bona fide tumor cells and with respect to a range of intermingling non-neoplastic cells, which also play a vital role in controlling the course of the disease. In fact, GB tumors contain an important infiltration of immune cells, especially myeloid cells, which are enriched [2, 3]. This robust myeloid-dependent immune response in GB is thought to increase tumor aggressiveness and lead to reduced survival [4, 5]. The tumor-associated myeloid cells are composed of microglia, i.e., resident immune cells of the central nervous system, and of peripherally recruited bone marrow-derived macrophages (BMDM). In mouse models of the disease, BMDM were shown, following disruption of the blood-brain barrier, to infiltrate these tumors only at later stages of their expansion [2, 6]. As further evidence of their essential role in glioma progression, removal of microglia, both in brain organotypic slices and genetic mouse models, inhibited glioma invasiveness [4, 5].

Targeting the resident microglia, as well as the peripherally recruited BMDM, in the tumor microenvironment has been proposed as an intervention to combat GB expansion. Myeloid cells depend on colony-stimulating factor-1 (CSF1) for differentiation and survival. Blockade of the CSF1 receptor (CSF1R) initially yielded promising results in a mouse model of GB with increased survival and regression of tumors [7]. However, GB tumors are shown to recur due to the acquisition by the myeloid cells of a resistance to CSF1R inhibition and the production of insulin growth factor 1 (IGF1) that instead drives tumor growth and survival. This could represent a mechanism that could account for the disappointing results observed in clinical trials of CSF1R blockade [8, 9].

During the course of the disease, microglia are recruited by the glioma cells as immune competent cells that potentially exhibit anti-tumoral effects, before they are reprogrammed into tumor-supporting cells by the same cancer cells, hence defining different microglial reactive states with unique targetable functions [4, 10, 11]. An additional therapeutic approach could focus on reprogramming microglia within the tumor microenvironment to restore their anti-tumoral functions. This approach relies on either impairing the acquisition of tumor-promoting capacity by microglia or on converting them into tumor-attacking cells, maintaining some of their intrinsic properties as innate immune cells. However, the molecular mechanisms that control the transition of microglia from an immune-reactive state with a unique gene expression profile and possible anti-tumoral properties into a phenotype with a distinct transcriptome that is associated with tumor-trophic functions in response to cues from glioma cells remain elusive. Transcriptional activation and repression are strongly influenced by and associated with changes in chromatin structure. In fact, epigenetic changes, including histone posttranslational modifications and DNA methylation, are important regulators of gene expression and have been involved in cell phenotype regulation as well as cellular reprogramming [12]. DNA methylation patterns placed by the DNA methyltransferase (DNMT) enzymes are reversible heritable marks conserved during cell division, which have been shown to be actively involved in cell reprogramming processes [13]. We sought to determine whether DNMT-mediated DNA methylation could be involved in glioma-induced microglial activation.

## Results

### Microglia are initially activated as immune-reactive cells in response to glioma stimulation

**Fig. 1:**
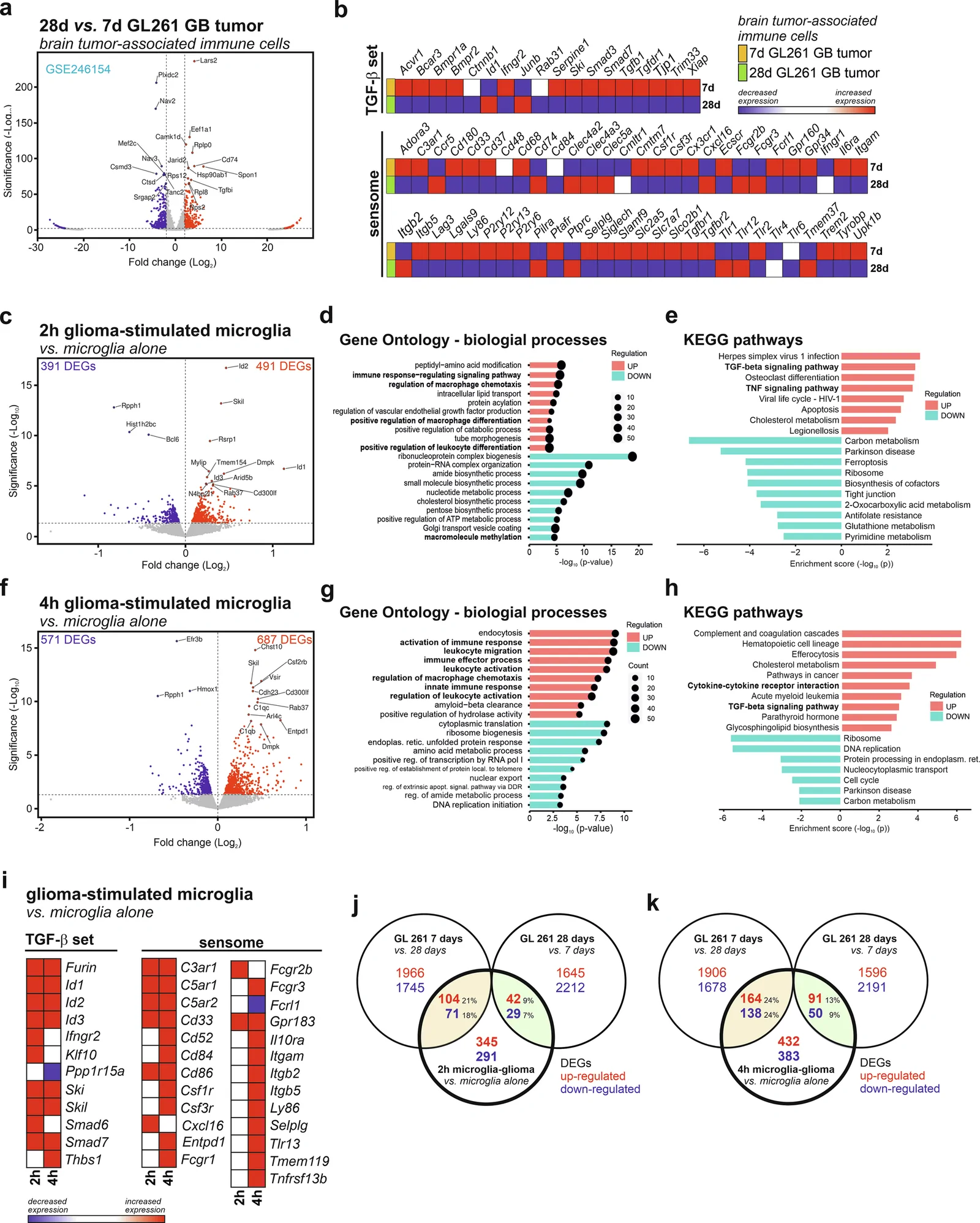
Early response of microglia to glioma cell stimulation is associated with an immune response-associated transcriptome. **a** Volcano plot representation of single nucleus RNA-seq transcriptomic source dataset comparing gene expression in tumor-associated immune cells, including microglia, obtained from mouse brains at 28 days versus 7 days post-implantation of GL261 GB cells (GEO accession number: GSE246154; differentially expressed genes (DEGs) were selected based on FDR < 0.05 and Fold Change ≥ 1.5). **b** The dataset was further analysed for gene expression related to the TGF-β-dependent microglial homeostasis and the microglial sensome gene sets. Volcano plot representations of the RNA-seq data comparing gene expression in BV2 microglia stimulated by soluble factors originating from C6 glioma cells (segregated coculture) versus microglia alone (monoculture) at 2 h (**c**) and 4 h (**f**) time points (*n* = 3, DEGs were selected based on a statistically significant *p*-value, *p* < 0.05). **d**, **e**, **g**, **h**, Gene ontology (GO) enrichment for biological processes (BP) (**d**, **g**) and KEGG pathway (**e**, **h**) analyses were performed separately for up- and down-regulated genes comparing DEGs in glioma-stimulated microglia versus microglia alone at 2 h (**d**, **e**) and 4 h (**g**, **h**) time points (The top 10 up- and down-regulated GO BP terms are displayed). **i** The dataset was further analysed for gene expression related to the TGF-β-dependent microglial homeostasis and the microglial sensome gene sets. Venn diagrams illustrating the overlap for the DEGs from in vitro C6 glioma-stimulated BV2 microglia versus BV2 microglia alone at 2 h (**j**) and 4 h (**k**) compared to the DEGs in immune cells collected from the 7 days versus 28 days, and vice versa, GL261 GB tumors (in vivo).

Glioma cells are reported to reprogram microglia, the brain resident myeloid immune cells, towards a tumor-supporting phenotype (reviewed in 4, 5). The preclinical immunocompetent syngeneic GB murine model based on the implantation of GL261 GB, IDH-wildtype cells into the brain parenchyma, recapitulates numerous characteristics of the human GB tumors [14]. Twenty-eight days post-intracerebral injection of GL261 GB cells, the microglia within the established neoplasm exhibit an upregulation of expression for genes involved in facilitating tumour growth and expansion, but a downregulation of expression for genes related to the transforming growth factor beta (TGF-β) signaling known to regulate their homeostatic functions, as well as for genes related to the microglial sensome coupled to their ability to sense challenges in their cellular environment [10, 15, 16]. We hypothesized that in response to stimulation by glioma cells, microglia could transit through distinctive states of activation(s) before acquiring a tumor-supportive phenotype. To test this possibility, we took advantage of publicly available single-nucleus RNA-sequencing (RNA-seq) transcriptomic data sets for tumor-associated immune cells, including microglia, obtained from mouse brains at 7 days and 28 days post-implantation of GL261 GB cells (Gene Expression Omnibus accession number: GSE246154). Tumors established from GL261 GB cells recapitulate numerous GB characteristics [14]. Comparison of the tumor-associated immune cells in the 28 days versus the ones in the 7 days GL261 GB tumors demonstrated that the immune cells, at those two different stages of tumor development, were characterized by distinct gene expression profiles. A volcano plot of differentially expressed genes (DEGs) between brain tumor-associated immune cells in the 28-day versus 7-days GL261 GB tumors is depicted in Fig. 1a. As previously described, the expression of gene sets related to the TGF-β-dependent microglial homeostasis and the microglial sensome were found to be down-regulated in the immune cells in the 28-day GL261 GB tumors [10]. In contrast, these two gene sets were found to be significantly enriched in the immune cells collected from the 7-days GL261 GB tumors (Fig. 1b). This in vivo transcriptomic data suggests that immune cells in the GB tumor microenvironment, such as the brain resident microglia, can acquire a distinctive activation state before they are turned into tumor trophic cells. After 24 h exposure to stimuli originating from C6 glioma cells, BV2 microglia are reported to exhibit a tumor-supportive phenotype able to promote the invasion capabilities of the cancer cells [11, 17]. To further investigate the possibility of the existence of a distinctive early glioma cancer cells-induced microglial activation state, BV2 microglia were exposed to C6 glioma, IDH1 wildtype cells, for limited time, i.e., 2 or 4 h, in a segregated coculture set-up, thereafter microglia were collected, and bulk RNA-seq was performed (Gene Expression Omnibus accession number: GSE314250) (Supplementary Data S1). This transcriptomic analysis showed DEGs between microglia collected from monoculture, used as a control, and those collected from segregated coculture with glioma cells. Volcano plots of DEGs between unstimulated microglia (monoculture) and glioma-stimulated microglia (coculture for 2 or 4 h) are depicted in Fig. 1c, f. Gene ontology (GO) term enrichment analysis for DEGs between unstimulated microglia and glioma-stimulated microglia unveils that up-regulated genes were associated with GO enrichment for biological processes related to immune response and regulation of macrophage differentiation and chemotaxis, as well as macromolecule methylation (Fig. 1d, g and Supplementary Data S2). KEGG (Kyoto Encyclopedia of Genes and Genomes) PATHWAY analysis that looks at DEGs enriched in selected signaling pathways highlighted a significant enrichment in microglia stimulated by glioma cells for the TGF-β signaling pathway (at both 2 h and 4 h) and tumour necrosis factor (TNF) signaling pathway (at 2 h) (Fig. 1e, h and Supplementary Data S3). Likewise, what was observed in vivo for immune cells at 7 days in the GL261 GB syngeneic mouse model, the expression of gene sets related to the TGF-β-dependent microglial homeostasis and the microglial sensome were found to be up-regulated in vitro in the 2 h and 4 h glioma-stimulated microglia (Fig. 1i). When the transcriptomes of the C6 glioma-stimulated BV2 microglia were compared to the transcriptomes of immune cells collected from GL261 GB tumors, the in vitro glioma stimulated microglia (both 2 h and 4 h) were shown to share higher numbers of DEGs with immune cells collected from the 7 days as compared to 28 days GL261 GB tumors (Fig. 1j, k). Collectively, these data support the use of short-term in vitro glioma-stimulated microglia as a model system to investigate the existence of a transient early microglial activation state not holding pro-tumoral properties.

### Early glioma cells-induced microglia activation is associated with a transient reduction in global microglial DNA methylation

**Fig. 2:**
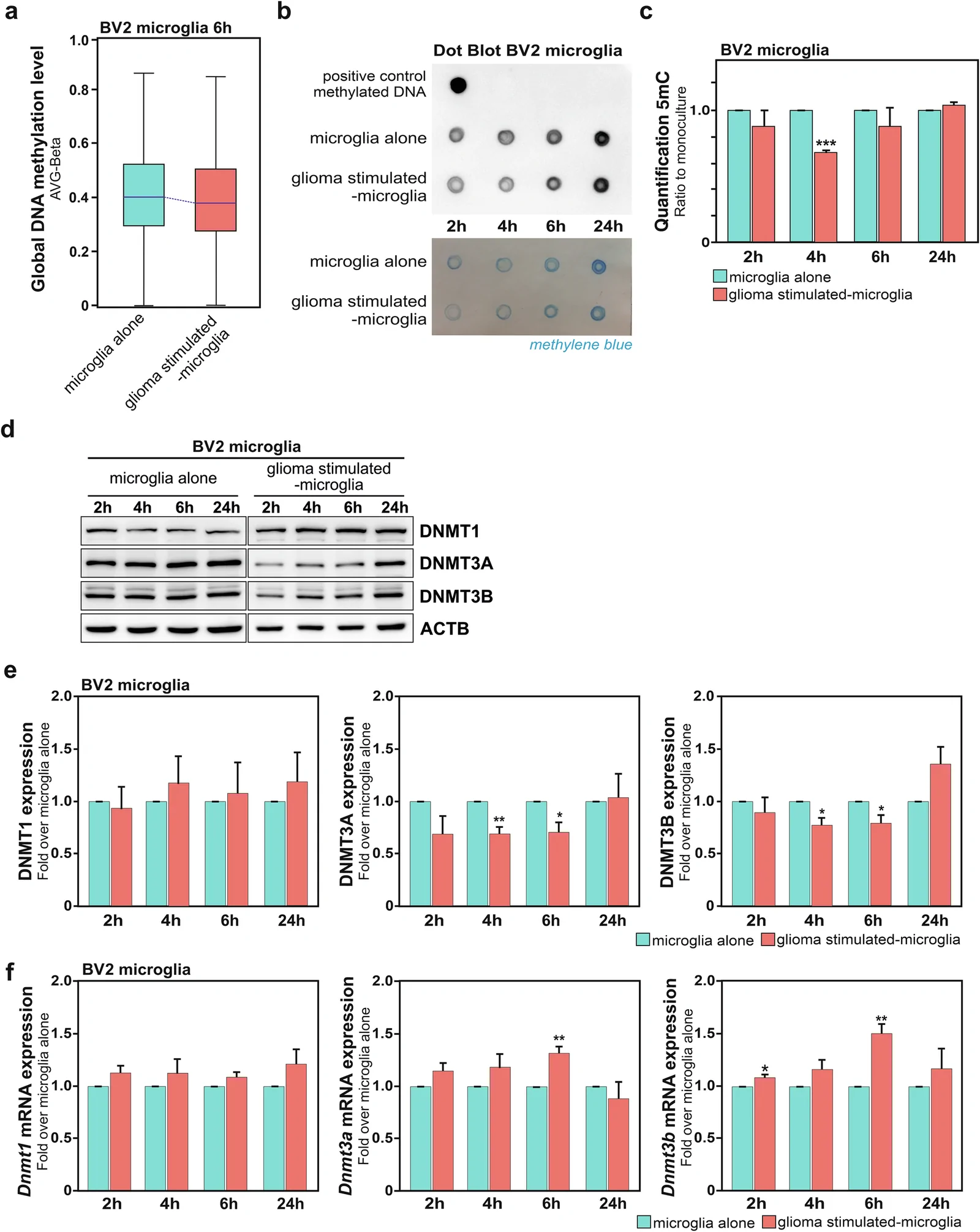
Transient reduction of global DNA methylation and DNMT3A/3B expression levels are observed in glioma-stimulated microglia*.* **a** High-throughput profiling of DNA methylation status at CpG islands performed in BV2 microglia after 6 h monoculture *versus* 6 h coculture with C6 glioma cells. The average methylation levels (Beta values) on the CpG islands for each sample obtained from the Illumina^®^ Infinium HumanMethylation450 BeadChip array analysis are shown as a box plot. Dot Blot analysis of 5-methylcytosine content (5-mC) in BV2 microglia after different time points in monoculture or coculture with C6 glioma cells, with corresponding methylene blue staining of the membrane below (**b**) with quantification (**c**), presented as fold of monoculture condition for each time point set as 1. Immunoblot analysis (**d**) and quantifications (**e**) of DNMTs proteins (DNMT1, DNMT3A and DNMT3B) and β-actin (ACTB) expression levels in BV2 microglia in monoculture or in coculture with C6 glioma cells at the indicated time-points. **f** RTqPCR analysis of microglial *Dnmt1*, *Dnmt3a* and *Dnmt3b* mRNA expression levels, using identical conditions as the ones described in (**a**). *P < 0.05, **P < 0.01, and ***P < 0.001 (pair comparisons, two-tailed Student’s *t* test) (**c**, **e**, **f**). Data are from three independent experiments (*n* = 3) and shown as mean ± sem.

Methylation of the 5′-position of cytosine residues, located adjacent to a guanine (CpG), is a reversible covalent modification of DNA resulting in the production of 5-methyl-cytosine (5-mC) that changes the biophysical characteristics of the DNA. In mammals, DNA methylation is recognized as an epigenetic process, known to efficiently influence gene expression at the transcriptional level [18]. Although the role of histone post-translational modifications, another epigenetic mechanism regulating transcriptional responses, in driving microglial polarization toward a tumor-supportive phenotype has been investigated [12], the potential contribution of DNA methylation remains largely unexplored. Given the rapid transcriptomic changes observed in microglia upon glioma stimulation, we hypothesized that DNA methylation might be involved. To investigate this, we used the above-described segregated coculture system and performed genome-wide DNA methylation profiling of BV2 microglia exposed to C6 glioma cells using the Illumina® Infinium Methylation 450 K BeadChip array. This platform assesses over 450,000 CpG sites at single-nucleotide resolution. Interestingly, we observed a modest decrease in the average beta value, a commonly used metric for DNA methylation, in glioma-stimulated microglia compared to unstimulated controls (Fig. 2a). Although this observation suggested a potential reduction in global DNA methylation, we recognized that it does not conclusively establish its occurrence in glioma-stimulated microglia. To further explore this possibility, we performed DNA dot blot analyses to quantify 5-mC content in BV2 microglia under coculture with C6 glioma cells and monoculture conditions at different time points. These experiments revealed a transient reduction in global DNA methylation following glioma stimulation: 5-mC levels were significantly decreased after 4 h of coculture but returned to baseline levels by 24 h (Fig. 2b, c).

### Decrease in microglial DNA methylation is associated with transient downregulation of DNMT3A and DNMT3B

The protein expression level of the main DNA methyltransferases (DNMTs), i.e., DNMT1, DNMT3A and DNMT3B, was investigated in BV2 microglia upon exposure to C6 glioma cells. Immunoblot analysis uncovered a significant reduction in the expression level of microglial DNMT3A and DNMT3B proteins between 4 and 6 h of segregated coculture with the glioma cells. Likewise, the observed decrease in microglial global DNA methylation, the reduction in DNMT3A and DNMT3B protein expression was transient, and their expression levels at 24 h in microglia from segregated glioma coculture were not different from microglia in monoculture (Fig. 2d, e). In those conditions, no significant reduction in the DNMT1 protein expression levels was observed (Fig. 2d, e). A significant increase in microglial *Dnmt3a* and *Dnmt3b* mRNA expression levels was observed after 6 h of microglia-glioma coculture that could be explained as a compensatory mechanism activated by the microglia to recover from the reduction in protein expression (Fig. 2f). Decreased microglial DNMT3A protein expression upon exposure to glioma derived soluble factors was further confirmed with additional segregated coculture combination with BV2 microglia exposed to GL261 GB cells (Extended Data Fig. 1a–c) and human HMC3 microglia exposed to human U87-MG, IDH1 wildtype, GB cells (Extended Data Fig. 2a–c).

Thereafter, we wanted to explore whether the observed transient DNMT3A downregulation in microglia cells is a distinctive characteristic of their activation by glioma cells, or a general mark for reactive microglia. To address this question, BV2 microglia were treated with lipopolysaccharide (LPS) or interleukin 4 (IL4) [19–21]. Neither LPS treatment nor IL4 treatment had an effect on the DNMT3A protein expression (Extended Data Fig. 3a, b). However, these treatments appear to affect microglial *Dnmts* mRNA expression levels (Extended Data Fig. 3c, d). Hence, the transient reduction in DNMT3A expression level and associated decrease in global DNA methylation are distinctive characteristics of the activation of microglia by glioma cells.

DNMT3A and DNMT3B are highly homologous proteins and share overlapping functions; however, they also exert distinct functions [22]. Of interest for the current study, a conditional *Dnmt3a* knockout mouse model revealed that large conserved domains of low DNA methylation, which cover regions enriched for genes involved in transcriptional regulation and therefore potentially with cell differentiation, are specifically maintained by DNMT3A [23]. In humans, *DNMT3A* haploinsufficiency causes DNA methylation defects that lead to abnormal gene expression and an impaired immune response of myeloid cells, as well as predisposition to myeloid malignancies [24]. In mice expressing a histone H3.3G34R amino acid substitution, i.e., a human germline mutation reported to cause severe neurodevelopmental disorders, DNMT3A recruitment to the chromatin is impaired, and an accumulation of disease-associated microglia coupled with the expression of innate immune-related genes is observed [25]. Collectively, the aforementioned observations and studies make DNMT3A an interesting candidate for the regulation of a specific microglial reactive state induced by early exposure to glioma cells.

### Transient reduction in DNMT3A DNA occupancy is observed in glioma-stimulated microglia at promoter regions of upregulated genes

**Fig. 3:**
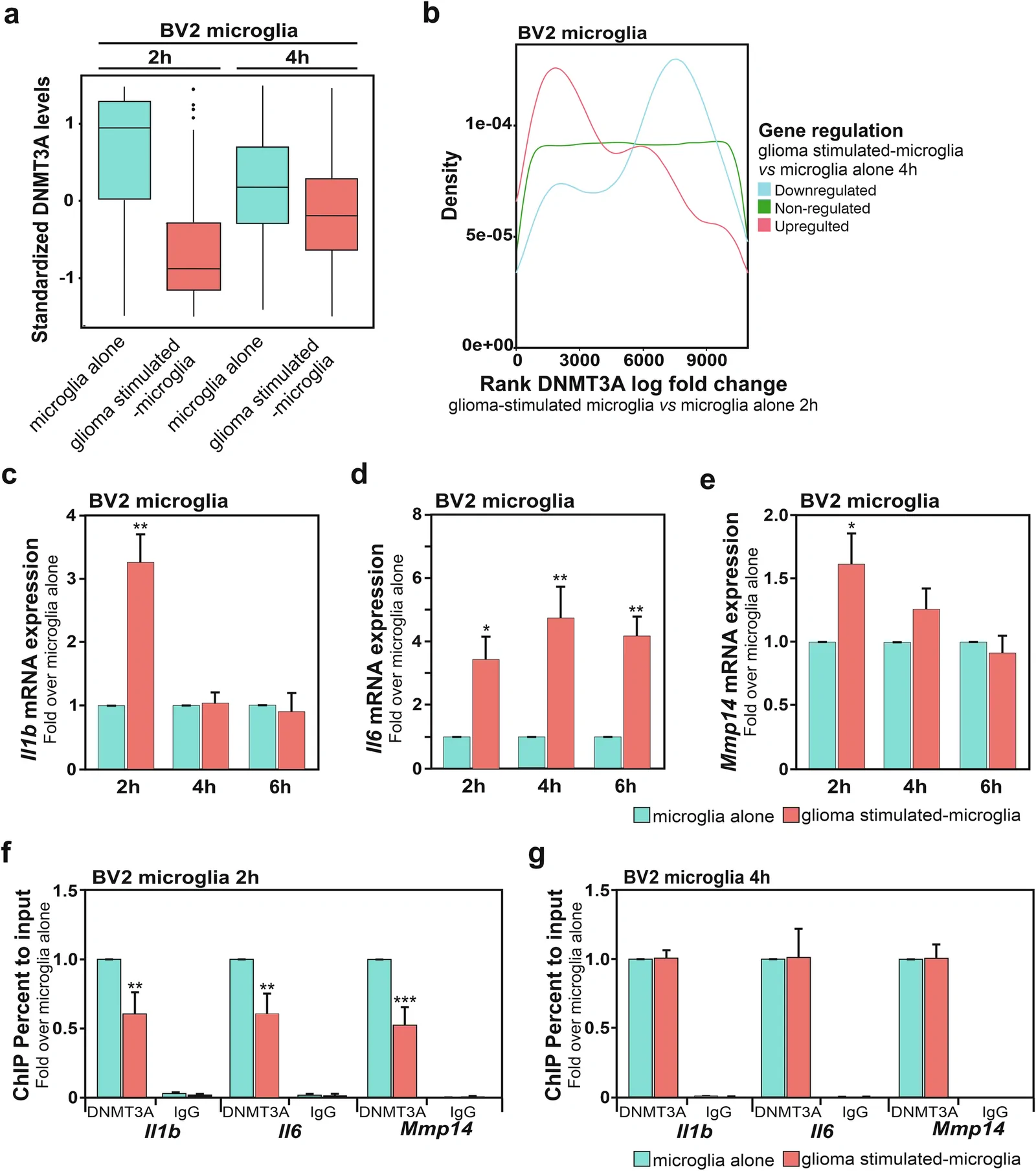
Transient reduction in DNMT3A DNA occupancy occurs in glioma-stimulated microglia at promoter regions of upregulated genes. **a** Box plot representation illustrating at the genome-wide level a general reduction in DNMT3A DNA occupancy in BV2 microglia stimulated for 2 or 4 h by C6 glioma cells; data obtained from DNMT3A ChIP-seq (*n* = 3). **b** Analysis of the relationship between DNMT3A DNA occupancy at 2 h in C6 glioma-stimulated BV2 microglia (as compared to BV2 alone) and gene expression at 4 h in C6 glioma-stimulated BV2 microglia (as compared to BV2 alone). The x-axis shows the DNMT3A DNA occupancy log fold change, ranked from the lowest occupancy (0) to the highest one (~10,000) when BV2 microglia were cocultured with C6 glioma compared to BV2 microglia monoculture at 2 h. Genes were then grouped according to their regulation (3 groups: downregulated, non-regulated, or upregulated) in C6 glioma-stimulated BV2 microglia at 4 h (as compared to BV2 alone). The density curves show how those gene groups are distributed in relation to the DNMT3A DNA occupancy. RTqPCR analysis of mRNA expression levels in BV2 microglia exposed to coculture with C6 glioma cells for 2, 4, and 6 h of *Il1b* (**c**), *Il6* (**d**), and *Mmp14/Mt1-mmp* (**e**). ChIP analysis of DNMT3A occupancy on the promoter region of *Il1b*, *Il6* and *Mmp14* genes in BV2 microglia after coculture with C6 glioma cells for 2 h (**f**) and 4 h (**g**) compared to their respective BV2 microglia monoculture. Data are presented as percent to input, and fold over monoculture condition set as 1. *P < 0.05, **P < 0.01, and ***P < 0.001 (pair comparisons, two-tailed Student’s *t* test) (**c**–**g**). Data are from three (*n* = 3, **g**), or four (*n* = 4, **c**, **d**, **f**), or six (*n* = 6, **e**) independent experiments and shown as mean ± sem.

DNMT3A chromatin immunoprecipitation sequencing (ChIP-seq) was performed to monitor at the genome-wide level the DNA binding of this enzyme upon glioma-induced microglia activation. This analysis revealed a general reduction in DNMT3A DNA occupancy in microglia stimulated for 2 h by glioma cells. This reduction in DNMT3A DNA binding was sustained at 4 h while at a lower level (Fig. 3a). Remarkably, the combined analysis of the RNA-seq and DNMT3A ChIP-seq microglial data sets showed that reduced DNMT3A DNA occupancy was observed (at 2 h) in genes whose expressions were found to be upregulated (at 4 h) upon stimulation by glioma cells (Fig. 3b).

Then, to get further insights into possible role for the displacement of DNMT3A from the chromatin into the regulation of an initial microglial reactive state induced by glioma cells, DNMT3A protein occupancy on the promoter regions of *Il1b, Il6* and *Mmp14 (*matrix metalloproteinase-14 (membrane-inserted), also known as *Mt1-mmp*) genes, microglial genes found to have an early regulation in response to glioma cell stimulation (Fig. 3c–e) was analyzed by ChIP in BV2 microglia collected from monoculture or from 2 and 4 h segregated coculture with C6 glioma cells (Fig. 3f, g). Induction of *IL1β/Il1β*, but not *IL6/Il6* gene expression, was also observed in BV2 microglia exposed to GL261 GB cells and HMC3 microglia exposed to U87-MG GB cells (Extended Data Fig. 1d and Extended Data Fig. 2d). In contrast, neither *Il1β* nor *Il6* gene expression was induced in BV2 microglia exposed to MN9D dopaminergic neuronal cells (Extended Data Fig. 4a, b) or C8-D1A astrocytes (Extended Data Fig. 4c, d).

The expression of MMP14 in microglia has been associated with the recruitment of microglia into the glioma tumor [26]. IL1β is a prototypic pro-inflammatory cytokine that plays key roles in both acute and chronic inflammatory responses [27]. IL6 is an interleukin reported to act as both a pro- and anti-inflammatory cytokine [28]. A robust decrease in DNMT3A protein occupancy at these 3 gene promoters was observed at the 2 h’ time-point in microglia collected from coculture with glioma cells, as compared to monoculture conditions (Fig. 3f). This difference in DNMT3A protein occupancy between these two conditions was lost at the 4 h’ time point, indicating a short and transient mechanism for the regulation of these microglial genes by DNMT3A (Fig. 3g).

Collectively, these experiments show that glioma-induced loss of DNMT3A-mediated DNA methylation at specific gene loci promotes their expression in microglia and contributes to the acquisition of a unique transcriptome associated with a pro-inflammatory and motile microglial cell phenotype.

### Downregulation of DNMT3A and concomitant upregulation of Il1 expression in primary microglia exposed to glioma cells

**Fig. 4:**
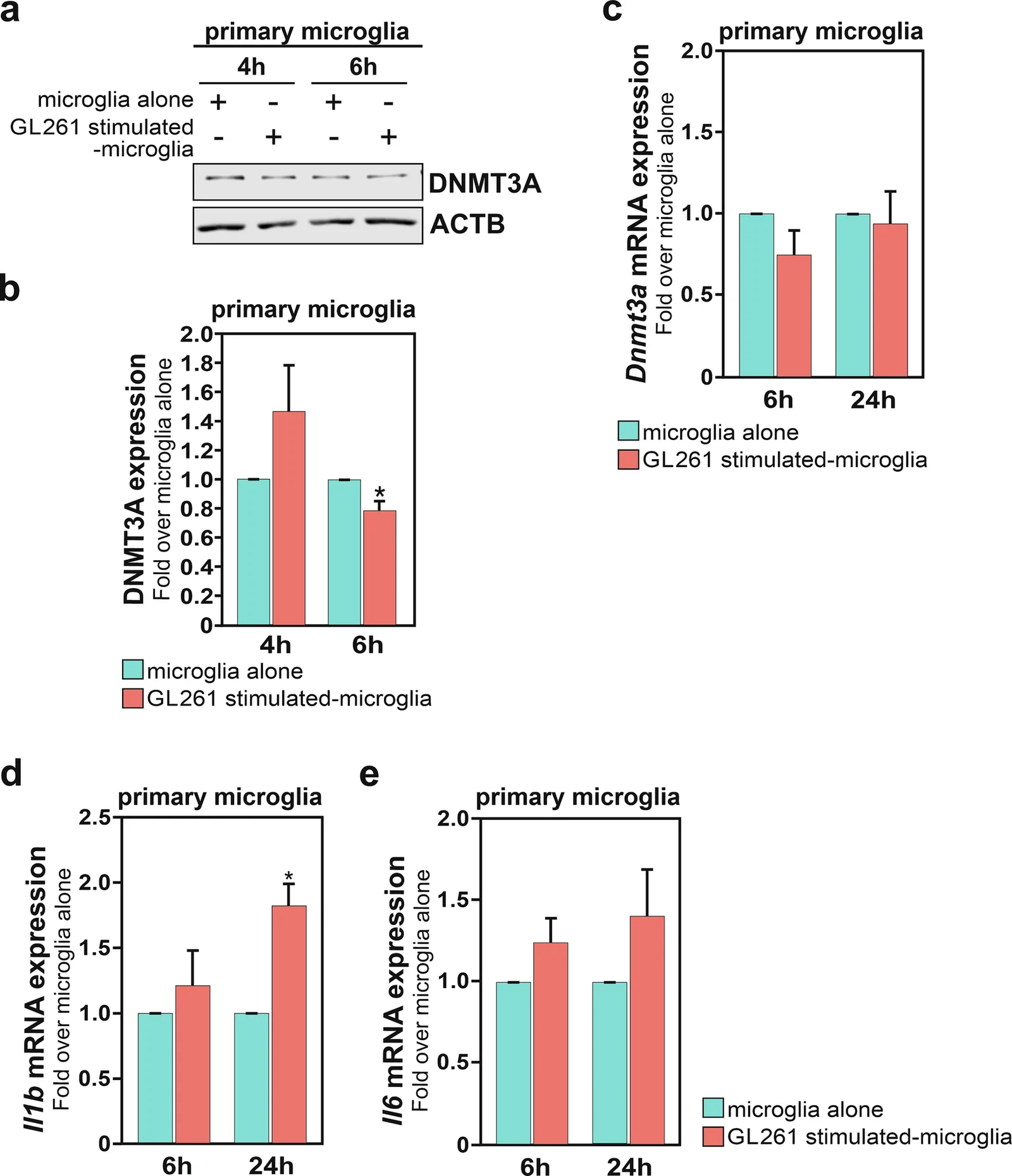
Down-regulation of DNMT3A, but up-regulation of *IL1b* expression in glioma-stimulated primary microglia. Immunoblot analysis (**a**) and quantifications (**b**) of DNMT3A and β-actin (ACTB) expression levels in primary microglia in monoculture or in coculture with GL261 GB cells at the indicated time-points. RTqPCR analysis of microglial *Dnmt3a* (**c**), *Il1b* (**d**) and *Il6* (**e**) mRNA expression levels, in primary microglia in monoculture or in coculture with GL261 GB cells at the indicated time-points, presented as fold of monoculture condition for each time point set as 1. *P < 0.05 (pair comparisons, two-tailed Student’s *t* test) (**b**–**e**). Data are from four independent experiments (*n* = 4, **b**–**e**) and shown as mean ± sem.

To validate the responses observed in BV2 microglia, we extended our analysis to primary microglia isolated from C57BL/6J mouse brains (Fig. 4). Protein expression of DNMT3A was assessed in primary microglia following exposure to GL261 glioblastoma (GB) cells. Immunoblot analysis revealed a significant reduction in DNMT3A protein levels after 6 h of segregated co-culture with GL261 cells (Fig. 4a, b). In contrast, *Dnmt3a* mRNA expression remained unchanged in primary microglia exposed to GL261 cells for up to 24 h (Fig. 4c). Notably, as in glioma-stimulated BV2 microglia, exposure to GL261 cells induced *Il1β* gene expression in primary microglia (Fig. 4d, e). Collectively, these findings confirm in glioma-stimulated primary microglia the key observations previously made in glioma-stimulated BV2 microglia.

### DNMT3A silencing in microglia promotes an anti-tumoral phenotype

**Fig. 5:**
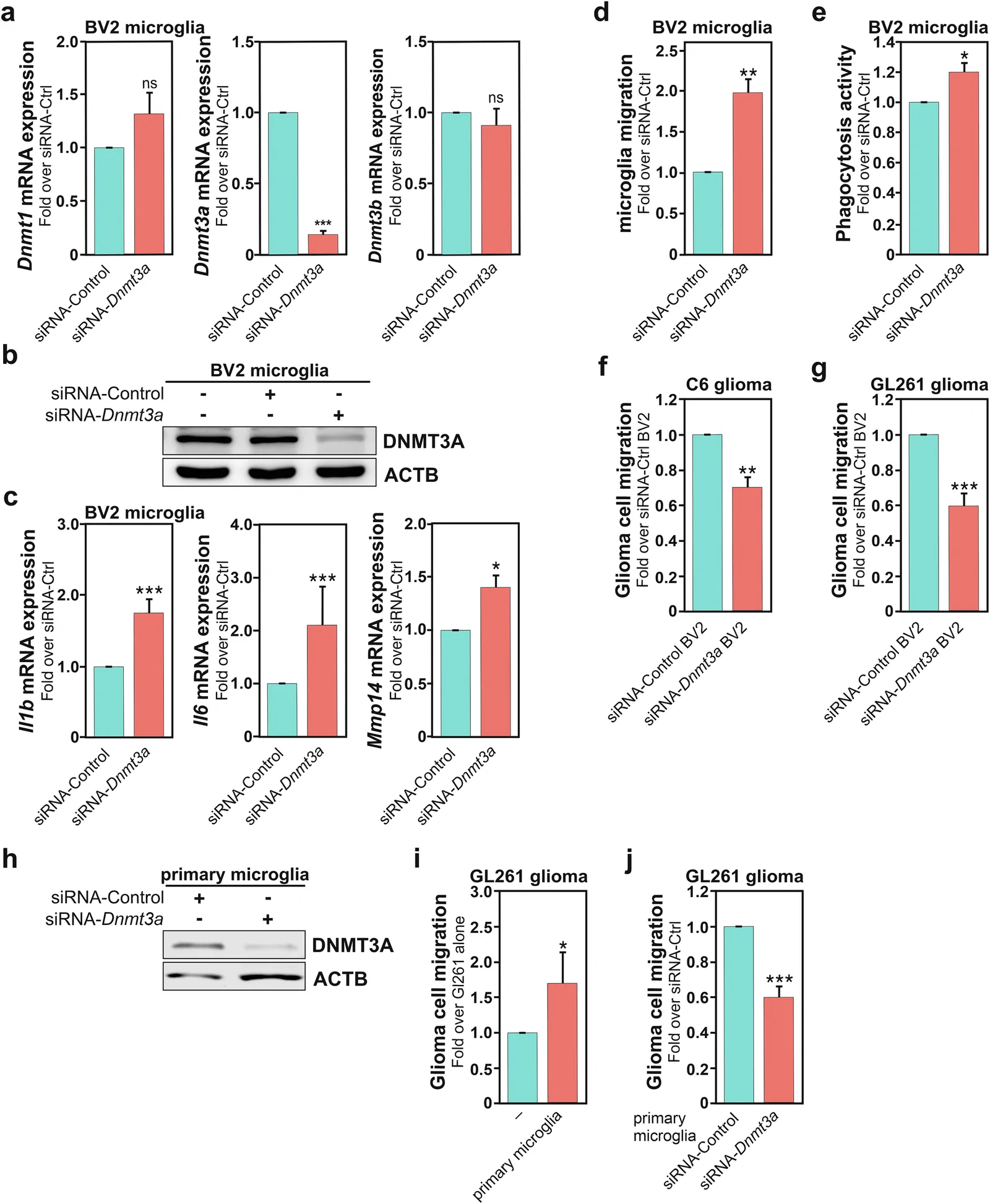
*Dnmt3a* silencing in microglia promotes an anti-tumoral phenotype in vitro. **a** RTqPCR analysis of *Dnmt1*, *Dnmt3a*, *Dnmt3b* mRNA expression levels in BV2 microglia transfected with a pool of siRNAs targeting *Dnmt3a* expression (siRNA-*Dnmt3a*), as compared to cells transfected with a pool of non-targeting siRNAs control (siRNA-Control/Ctrl, negative control), set as 1. **b** Immunoblot analysis of DNMT3A and β-actin (ACTB) expression levels in control BV2 microglia (-, mock transfected cells) or BV2 microglia transfected with a pool of non-targeting siRNAs control (siRNA-Control/Ctrl) or with a pool of siRNAs targeting *Dnmt3a* expression (siRNA-*Dnmt3a*). **c** RTqPCR analysis of *Il1b, Il6*, and *Mmp14*, mRNA expression levels in BV2 microglia transfected with siRNA-*Dnmt3a* compared to cells transfected with siRNA-Control, set as 1. **d** BV2 microglia transfected with siRNA-*Dnmt3a* exhibit increased cell migration capability when compared to cells transfected with non-targeting siRNA-Control. **e** Phagocytosis activity of BV2 microglia is reduced in cells transfected with siRNA-*Dnmt3a* compared to cells transfected with siRNA-Control. C6 glioma cells (**f**) or GL261 glioma cells (**g**) migration toward BV2 microglia is reduced when BV2 cells are transfected with siRNA-*Dnmt3a* compared to microglial cells transfected with siRNA-Control. **h** Immunoblot analysis of DNMT3A and β-actin (ACTB) expression levels in primary microglia transfected with a pool of non-targeting siRNAs control (siRNA-Control/Ctrl) or with a pool of siRNAs targeting *Dnmt3a* expression (siRNA-*Dnmt3a*). GL261 glioma cells migration toward primary microglia (**i**) is reduced when primary microglia are transfected with siRNA-*Dnmt3a* compared to primary microglia transfected with siRNA-Control (**j**). *P < 0.05, **P < 0.01, and ***P < 0.001 (pair comparisons, two-tailed Student’s *t* test) (**a**, **c**–**g**, **i**, **j**). Data are from three (*n* = 3, **a**, **d**, **f**), four (*n* = 4, **g**), five (*n* = 5, **e**), seven (*n* = 7, **c**(*Mmp14*)), eight (*n* = 8, **c** (*Il6*)) or nine (*n* = 9, **c**(*Il1b*)) independent experiments and shown as mean ± sem.

It is interesting to hypothesize that the observed downregulation of microglial DNMT3A and associated transcriptional effects, in response to a glioma stimulus, contributes to the polarization of the microglia cells toward a phenotype that could exert anti-tumoral functions. We therefore decided to assess the role of DNMT3A in the activation of BV2 microglia by knocking down the expression of endogenous *Dnmt3a* using a pool of small interfering RNAs (siRNAs), thereby mimicking the effect glioma cells have on microglial DNMT3A expression. This *Dnmt3a*-targeting siRNAs pool led to robust downregulation of the expression of the *Dnmt3a* mRNA (but did not affect *Dnmt1 and Dnmt3b* mRNAs expression) (Fig. 5a), as well as the expression of DNMT3A protein in BV2 microglia (Fig. 5b). RT-qPCR analysis revealed that *Dnmt3a* gene silencing in BV2 microglia was associated with a significant increase in *Il1b* mRNA expression levels (Fig. 5c). It is worth noting that co-culture of BV2 microglia with C6 glioma cells or GL261 GB cells did not alter the viability of these cells, as assessed by flow cytometric Annexin V/Propidium Iodide staining (Extended Data Fig. 5a, b). Similarly, transfection of BV2 microglia with either a *Dnmt3a*-targeting siRNA pool or a non-targeting siRNA control pool did not affect BV2 cell viability (Extended Data Fig. 5c).

Then, in a transwell system, we studied the effects microglial *Dnmt3a* gene silencing has on the capability of microglia to migrate, and the ability of these reactive cells to promote the migration of glioma cells. Whereas the inhibition of microglial DNMT3A expression appears to stimulate the migration capability of BV2 microglia (Fig. 5d), it reduces their aptitude to encourage the migration of C6 glioma cells and GL261 GB cells (Fig. 5f, g). Likewise, the repression of DNMT3A expression in primary microglia using the *Dnmt3a*-targeting siRNAs pool (Fig. 5h) reduced their ability to promote the migration of GL261 GB cells (Fig. 5i, j). In accordance with their increased migration capability, *Mmp14* gene expression was found to be increased upon repression of *Dnmt3a* expression in the BV2 microglia (Fig. 5c). Knockdown of *Dnmt3a* gene expression in BV2 microglia also promoted their phagocytic capacity as revealed by phagocytosis assay with beads (Fig. 5e). Repression of *Dnmt1* or *Dnmt3b* expression in BV2 microglia using siRNAs pools (i.e., siRNA-*Dnmt1* or siRNA-*Dnmt3b*) did not mimic the effect observed upon *Dnmt3a* silencing (Extended Data Fig. 6a–e).

Collectively, these observations advocate that the sustained repression of DNMT3A expression in microglia is sufficient to promote an immune and inflammatory phenotype (similar to the one observed in microglia upon short exposure to glioma cells).

### In vivo, DNMT3A-deficient microglia reduce glioma tumor growth

Based on our finding that the regulation of DNMT3A is involved in the control of a gene expression profile in microglia associated with inflammatory and immune reactive responses, which can potentially exert anti-tumoral functions, it is attractive to speculate that repressing its expression in microglia could provide us with cells that exert beneficial, anti-tumoral effects in the context of glioma tumors.

**Fig. 6:**
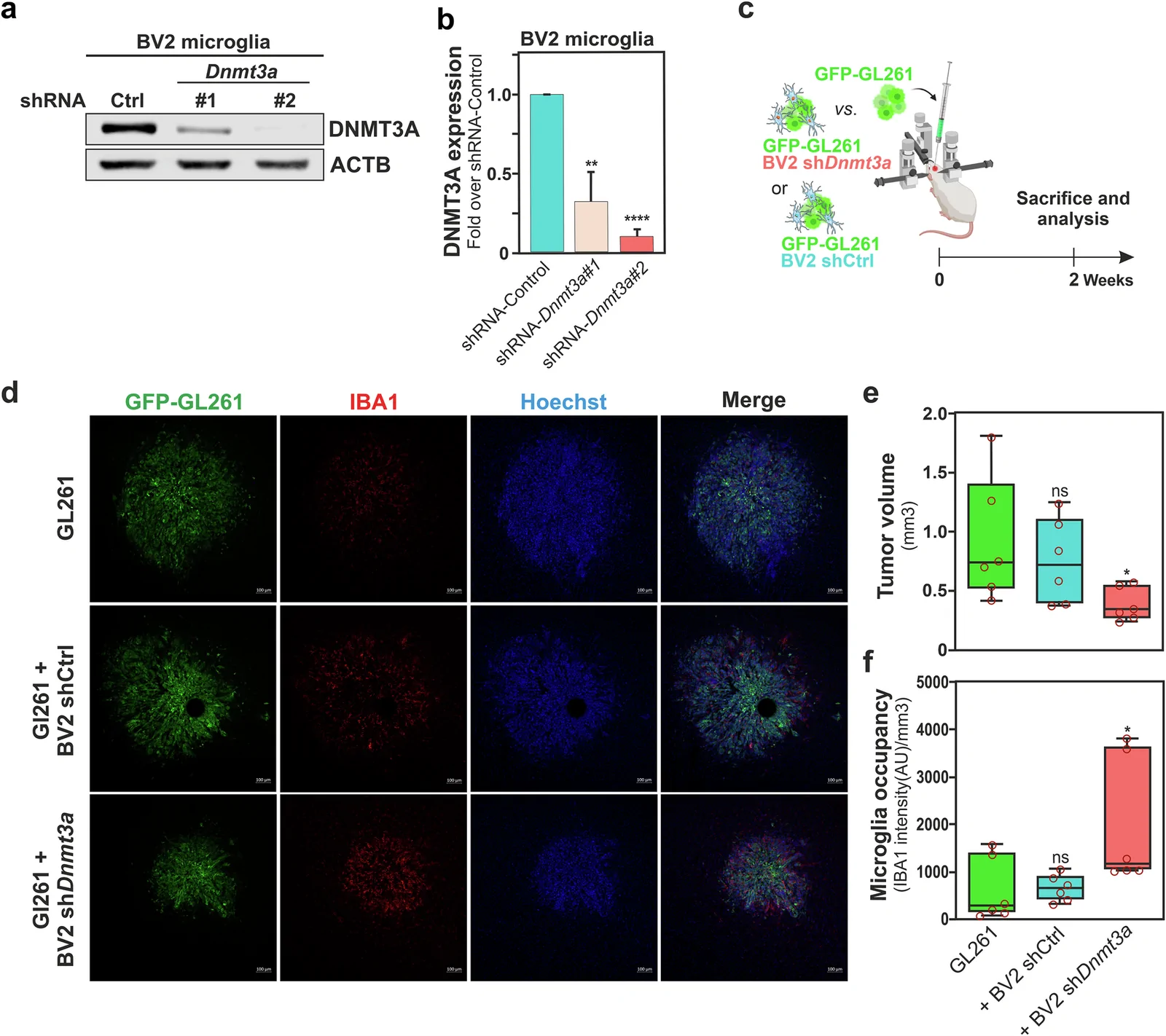
*Dnmt3a* silencing in microglia promotes an anti-tumoral phenotype in vivo. Immunoblot analysis (**a**), with quantifications (**b**), of DNMT3A and β-actin (ACTB) expression levels in BV2 cell lines generated to express constitutively a vector encoding for shRNA targeting *Dnmt3a* expression (2 different lentiviral clones #1 and #2 are depicted) or an empty vector used as control (Ctrl). **c** Scheme representing the workflow of the in vivo experiments performed with the co-injection in mouse brain of shRNA Control/Ctrl BV2 microglia or shRNA *Dnmt3a* BV2 microglia with GFP-GL261 glioblastoma cells. **d** Confocal immunofluorescence imaging showing GFP-GL261 glioblastoma cells (green), and IBA1-expressing microglia (red) in mouse brain tissues 2 weeks after the intracranial injection GL261 cancer cells alone, a mix with shRNA Control BV2 microglia or a mix with shRNA *Dnmt3a* BV2 microglia. Hoechst was used for nuclear counterstain (blue). Quantification of the tumor sizes (**e**) and microglial occupancy (**f**) for the conditions described in (**d**). *P < 0.05, **P < 0.01, and ***P < 0.001 (pair comparisons of each shRNA *Dnmt3a* to shRNA Control, two-tailed Student’s *t* test (**b**), or one-way ANOVA with Dunnett’s multiple comparisons test (**e, f**)). Data are from three independent experiments (*n* = 3, **b**) or six mice per group (*n* = 6, **d**–**f**). Data are shown as mean ± sem (**b**), or box plot with data point for individual mice depicted as a circle (**e**, **f**).

Viral delivery of small hairpin RNA (shRNA) targeting *Dnmt3a* was used for the establishment of BV2 microglia-derivatives with stable knockdown of DNMT3A expression (Fig. 6a, b). A *Dnmt3a* shRNA-expressing microglial clone (i.e., BV2 shRNA *Dnmt3a*#2) that exhibited a 90% reduction in DNMT3A protein expression, as compared to a microglial clone infected with the lentiviral empty vector control shRNA (BV2 shRNA Control), was selected for further experimentation. To examine the physiological relevance of our findings, we performed in vivo experiments and injected GFP-expressing GL261 GB cells or co-injected GL261 GB cells together with BV2 shRNA *Dnmt3a* or BV2 shRNA Control into the brain of young C57BL6/J mice (Fig. 6c). This syngeneic transplant tumor model in immunocompetent mice has been shown to exhibit limited infiltration by peripheral monocytes or macrophages at the time points used [6]. Immunohistochemical analysis of brain tissue 2 weeks post-transplantation revealed a significant reduction in GFP-GL261 GB tumor sizes in mice co-injected with BV2 shRNA *Dnmt3a*, but not in those co-injected with BV2 shRNA Control, as compared to those injected with the GFP-GL261 GB cells alone (Fig. 6d, e). Furthermore, IBA1 immunofluorescence intensity within the tumor volume, used as a proxy for microglial occupancy, was increased in tumors from mice co-injected with BV2 shRNA Dnmt3a, but not in those co-injected with BV2 shRNA Control, compared to tumors from mice injected with GFP-GL261 cells alone (Fig. 6d, f). Collectively, these observations suggest that repression of DNMT3A in microglia promotes an effective anti-tumoral phenotype in these myeloid cells.

### In vivo, Dnmt3a antisense oligonucleotides activate microglia and reduce glioma tumor growth

**Fig. 7:**
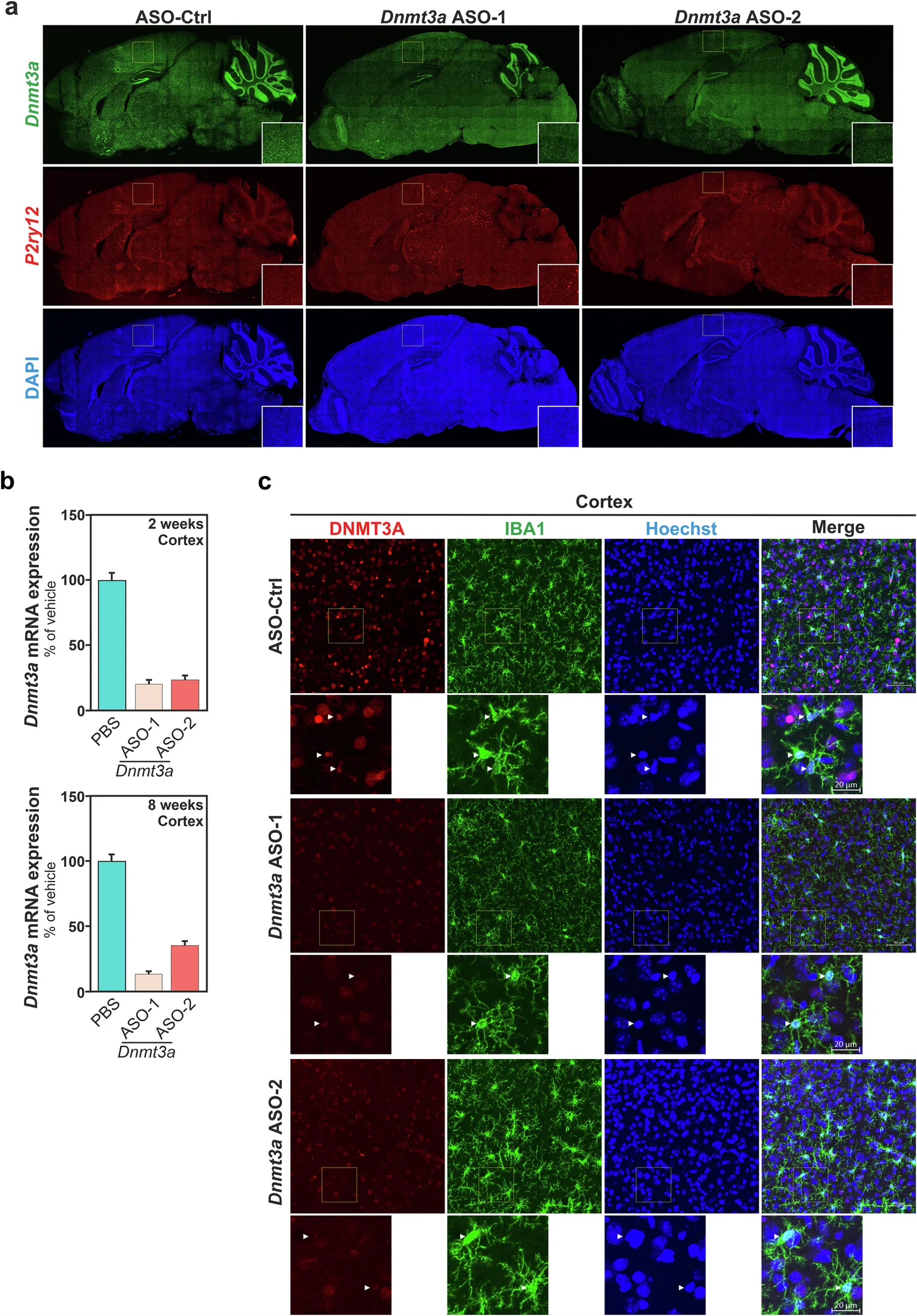
ASOs targeting *Dnmt3a* efficiently reduce *Dnmt3a* mRNA and DNMT3A protein expression levels in vivo. **a** Confocal analysis of *Dnmt3a* (green) and *P2ry12* (red, used as a microglial marker) mRNA expressions by RNAscope in the brains of mice 2 weeks post injection of non-targeting ASO (ASO-Ctrl) or ASOs targeting *Dnmt3a* (ASO-1 and ASO-2). DAPI (blue) was used as a nuclear counterstain. The yellow squares indicate the regions magnified and presented on the right-hand side of each condition. **b** RTqPCR analysis of *Dnmt3a* mRNA expression level in the cortex of mice injected with PBS (used as control) or with ASOs targeting *Dnmt3a* (ASO-1 and ASO-2) and sacrificed at 2 weeks or 8 weeks after injection, ratio to vehicle, average of two independent experiments. **c** With similar conditions as described in panel a, confocal immunofluorescence analysis of DNMT3A and IBA1 (used as a microglial marker) protein expressions (red) in the cortex mice brain area. Hoechst (blue) was used as nuclear counterstain. The yellow squares indicate the regions magnified and presented below of each condition. (Scale bar, 50 µm, in zoom, 20 µm).

Encouraged by these promising results, and to gain direct evidence that reducing microglial *Dnmt3a* expression in vivo could lead to a reduction in GB tumor growth, we made use of the antisense oligonucleotides (ASOs) developed by Ionis® Pharmaceuticals to target mouse *Dnmt3a* in a highly specific manner. RNA antisense therapies, including for CNS disorders (e.g., Ulefnersen, Tofersen, Zilganersen, ION859/BIIB094), have shown promising preclinical settings as well as in clinical trials (ClinicalTrials.gov) [29–31]. Two different mouse-specific *Dnmt3a*-targeting ASOs (referred to as *Dnmt3a*-ASO-1 and *Dnmt3a*-ASO-2) were developed and tested in vivo. Thereafter, the efficiency of these ASOs was tested at 2 and 8 weeks after injection in the right ventricle of C57BL6/J mice brains and showed a strong reduction in global *Dnmt3a* expression in the cortex of the injected mice (Fig. 7b). Both *Dnmt3a*-targeting ASOs were found to be safe based on delayed functional observational battery (FOB) and RTqPCR analysis of neuroinflammation markers. Further analyses using RNAscope and immunofluorescence confocal imaging confirmed the reduction of *Dnmt3a* mRNA and DNMT3A protein levels 2 weeks post-injection of either ASO-1 or ASO-2, as compared to a control non-targeting ASO (ASO-Ctrl) (Fig. 7a, c).

**Fig. 8:**
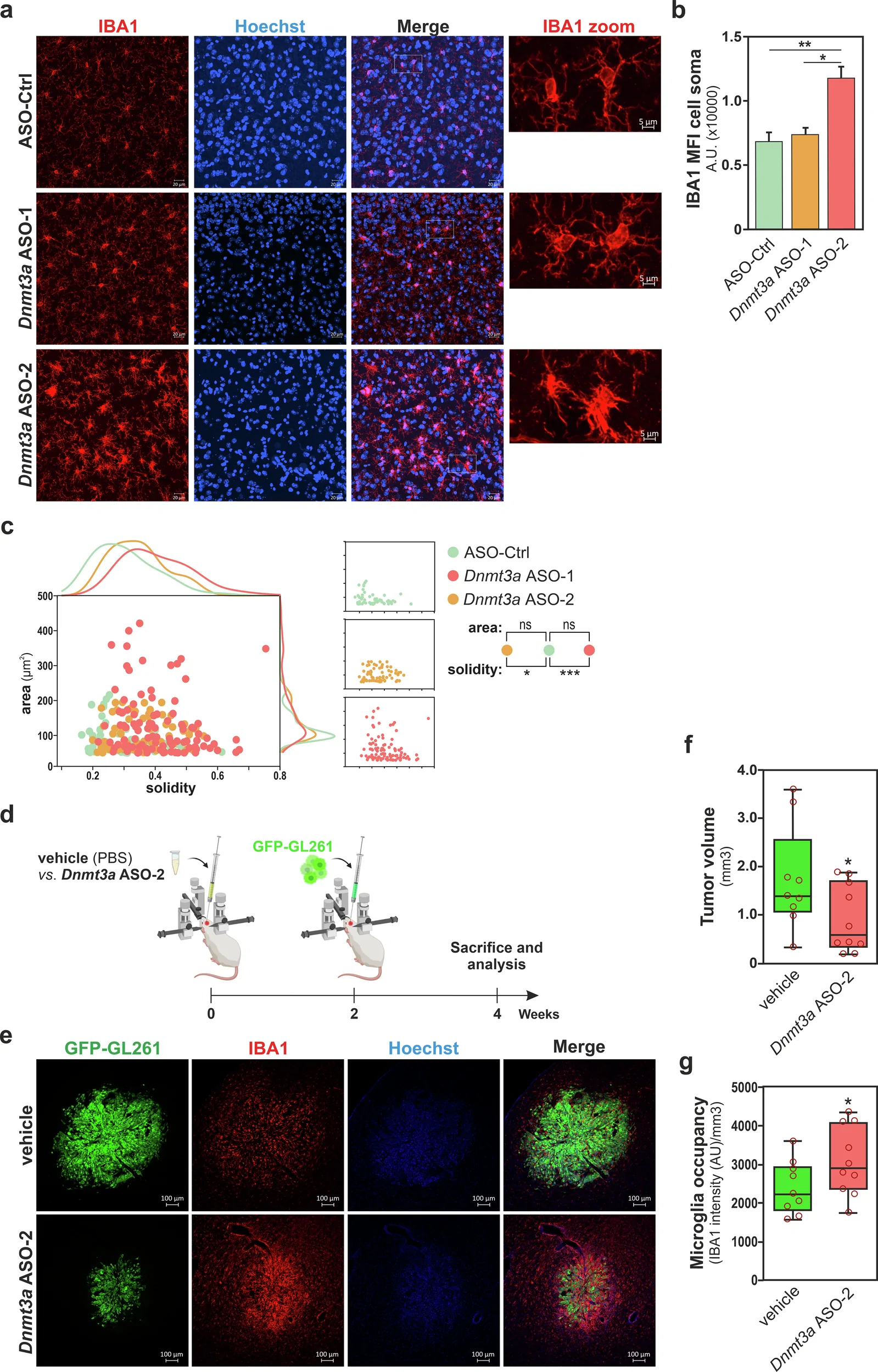
ASOs targeting *Dnmt3a* induce microglia activation and decrease glioma tumor growth in vivo. **a** Confocal immunofluorescence analysis of microglial IBA1 expression (red) and Hoechst-nuclear couterstaining (blue) in brain tissues of mice injected with ASO-Ctrl or ASO targeting DNMT3A (ASO-1 and ASO-2). The white rectangles indicate the regions magnified and presented on the right-hand side of each condition, which illustrate microglia harboring different shapes depending on the treatment (referred to as IBA1 zoom). (Scale bars, 20 µm, 5 µm in zoom). **b** Quantification using IMARIS software of the IBA1 intensity (mean per cell) in microglia from mice treated with a non-targeting control ASO (ASO-Ctrl) or ASOs targeting *Dnmt3a* expression (ASO-1 and ASO-2). **c** Multiparametric morphologic analysis of individual microglia from mice treated with a non-targeting control ASO (ASO-Ctrl) or ASOs targeting *Dnmt3a* expression (ASO-1 and ASO-2), assessing cell area and solidity. **d** Scheme representing the workflow of the in vivo experiments performed with the GFP-GL261 GB mouse model presented in (**e**–**g**). **e** Confocal immunofluorescence imaging of tumors formed 2 weeks post injection of GFP-GL261 glioma cells (green) in mice previously treated with PBS, or ASO-2 (as illustrated in c) together with an immunostaining for the microglial marker IBA1 (red) and Hoechst used as nuclear counterstain (blue). Quantification of 2-week-old GL261 tumor volume (**f**) and microglia occupancy (**g**) in mice injected with PBS or ASO-2, 2 weeks prior to the injection of GFP-GL261 cancer cells. (Scale bars 100 µm). *P < 0.05, **P < 0.01, and ***P < 0.001 (one-way ANOVA followed by Tukey’s (**b**) or Dunnett’s post-hoc test for comparisons against controls (**c**), pair comparisons, two-tailed Student’s *t* test (**f, g**). Data are from the average of 50 cells per brain of 3 mice per group (*n* = 3, **b**), from at least 60 individual microglia obtained from input images from 4 mice per group (*n* = 4, **c**), or nine (PBS) or ten (*Dnmt3a* ASO-2) mice per group (*n* = 9/10, **e**, **f**). Data are shown as mean ± sem (**b**), or box plot with data point for individual mice depicted as a circle (**f, g**).

Analysis of microglia in the brains of mice injected with ASO targeting *Dnmt3a* highlighted that the reduction of *Dnmt3a*/DNMT3A expression induces a microglial reactive state. Indeed, microglia in brains injected with the *Dnmt3a*-ASO-2 displayed an amoeboid shape, characteristic of activated microglial cells, associated with a significant increase of ionized calcium binding adaptor molecule 1 (IBA1, also known as allograft inflammatory factor 1, AIF1) immunofluorescence intensity (Figs. 7c and 8a, b). Multiparametric morphologic analysis of individual microglia, assessing parameters such as cell area and solidity using *CellProfiler™*, revealed a significant increase in solidity in microglia from brains injected with both *Dnmt3a*-targeting ASOs compared to those injected with ASO-Control. This morphological alteration was more pronounced following *Dnmt3a*-ASO-2 treatment (Fig. 8c). Additional brain immunofluorescence confocal imaging revealed an increase in staining intensity for galectin-3 (LGALS3), whose expression is associated with a microglial inflammatory response [32], 2 weeks after the injection of ASO-2 compared to ASO-Control; further validating the presence of an immune activated microglial phenotype after *Dnmt3a* knockdown (Extended Data Fig. 7a).

Finally, we investigated the potential anti-tumoral properties of our ASO targeting *Dnmt3a* in an in vivo GB tumor mouse model. Based on the above observations that in vivo the *Dnmt3a*-ASO-2, as compared to *Dnmt3a*-ASO-1, (i) had a better acute tolerability, and (ii) was more effective in promoting a microglial reactive state, this *Dnmt3a*-targeting ASO was selected for further in vivo investigations. Two weeks prior to the transplantation of GFP-GL261 GB cells into the brain of C57BL6/J mice, those mice were injected with *Dnmt3a*-ASO-2, or with PBS, i.e., vehicle for ASO injection, used as control (workflow in Fig. 8d). Two weeks post-injection of the GFP-GL261 GB cells, mice were sacrificed, and the presence of microglia as well as tumor size were investigated by immunofluorescence confocal imaging (Fig. 8e). IBA1 used to detect the microglial cell population was observed to be more prominently expressed within tumors of *Dnmt3a*-ASO-2 injected mice compared to PBS-injected ones (Fig. 8e, g). In addition, detection of the GFP-GL261 GB cells after ASO treatment revealed a significant reduction in tumor growth in mice injected with the *Dnmt3a*-ASO-2 compared to PBS-injected animals (Fig. 8e, f). Importantly, the injection with the ASO-Ctrl did not yield the effects observed with *Dnmt3a*-ASO-2 on both GFP-GL261 GB tumor growth and tumor microglia occupancy (Extended Data Fig. 7b–d). In summary, specific ablation of microglial DNMT3A negatively affects their tumor-supporting function in favor of anti-tumoral properties, and an RNA-based antisense strategy that selectively counteracts *Dnmt3a* expression led to both microglia activation and reduced glioma expansion in vivo.

## Discussion

A widespread view in the literature is that glioma cells recruit microglia, resident immune cells within their brain microenvironment, and convert them into tumor-supporting cells. During that process, the tumor cells are reported to shut down their immune properties and stimulate the microglia to exert tumor cell trophic effects instead, thereby contributing to a pro-invasive and immunosuppressive tumor environment [4, 5]. At the transcriptomic level, glioma cells are reported to hijack microglial gene expression to promote tumor growth and expansion [10]. In murine GB models, advanced tumors (e.g., 4 weeks post intracranial injection of GL261 GB cells in C57BL/6 mice brains, syngeneic transplant in an immunocompetent environment) are characterized by the downregulation of the expression of microglial genes involved in the sensing and dismissal of tumor cells. At the same time, the microglial expression of genes involved in facilitating tumor spread and expansion is upregulated [2, 3, 10]. Likewise, the analysis of transcriptomic data sets obtained from human GB biopsies reveals a proliferative and anti-inflammatory phenotype for the tumor-associated microglia that preferentially reside at the leading edge of tumor infiltration [33–35].

Our findings challenge this view, as it appears that glioma cancer cells initially promote a microglial phenotype, which is associated with a gene expression profile associated with both pro-inflammatory and immune responses. However, this immune-responsive phenotype is temporary, and the microglia, in response to cancer cell stimulation, turn into persistent tumor-supportive cells. Supporting the existence of a transient glioma cancer cell-induced microglial immune reactive state, in segregated cocultures performed with human CHME5 or murine BV2 microglial cells together with murine C6 glioma cells, transitory modifications of microglial morphology and metabolism were observed and found to be linked to a concomitant transitory increase of phagocytic capability; features that gradually disappeared with prolonged glioma stimulation [36]. Further evidence comes from in vivo observations made with the GL261 GB mouse model, at times where microglia recruitment and tumor growth are observed (i.e., 1 and 2 weeks post brain implantation of the GL261 GB cells) [6]. Indeed, at those earlier stages of GB development, microglia outside the tumor mass were shown to express the active caspase-3 that has been linked to a microglial proinflammatory phenotype, whereas microglia found within the core of the tumor showed an inhibition of basal caspase-3 activity associated with the polarization of microglia toward a tumor-supportive phenotype [11, 19]. Hence, delineating the molecular mechanisms that control the microglial activation via a transient, yet potentially anti-tumoral phenotype, in response to cues from glioma cells is of considerable interest.

The cellular reprogramming of microglia toward a particular phenotype with unique functions requires the acquisition of a new gene expression signature, in the context of an unchanging genomic sequence. Epigenetic changes, including histone modifications, microRNA expression, and DNA methylation, are important modulators of gene expression and have been involved in cell phenotype regulation and reprogramming and are therefore part of the mechanisms regulating cellular plasticity. In contrast to the extensive investigations made on the effects of histone-modifying enzymes and associated histone posttranslational changes, or of microRNA on microglial gene expression, the regulation of gene expression by DNA methylation remains poorly explored in those cells (for review see 12).

DNA methylation catalyzed by DNMTs is a relatively stable, yet reversible, epigenetic modification that plays important roles in the establishment and stabilization of cellular phenotypes by maintaining gene expression profiles [13]. In fact, DNA methylation can be conserved during cell division and is mostly associated with sustained transcriptional repression. This heritable chromatin mark has been involved in various cell reprogramming processes [37]. Hence, DNA methylation mediated by DNMTs is often viewed as a mediator of long-term epigenetic effects. However, we uncovered that glioma-induced microglia activation is associated with the transient displacement of microglial DNMT3A from chromatin sites, reduction in DNA methylation, and the induction of the expression of genes that confer to these cells a temporary transcriptome profile reflecting an immune-reactive phenotype.

The involvement of a rapid yet transient reduction in DNA methylation in reprogramming microglia toward a temporarily activated state may represent an atypical epigenetic stress response; however, similar mechanisms have previously been reported in other cell types undergoing differentiation. For example, a transient DNA demethylation driven by a loss of DNA methyltransferase proteins is a molecular characteristic of the mouse embryonic stem cell subpopulation that cycles through the so-called MERVL/Zscan4 cell state [38]. In addition, active DNA demethylation appears to be a common feature of the response of human innate immune cells, e.g., T cells, natural killer cells, neutrophils, and macrophages, facing an infectious agent [39–42]. Our findings reveal that, in response to glioma stimulation, a transient DNA demethylation, driven by reduced DNMT3A chromatin occupancy, is associated with the induction of a gene expression profile reflecting pro-inflammatory and immune responses, and thus potentially anti-tumoral functions in microglia. These results support the rationale for targeting microglial DNA demethylation-mediated signaling pathways as a therapeutic strategy against gliomas.

DNA demethylating agents, de facto DNMTs inhibitors, such as Azacytidine and Decitabine (5-aza-2′-deoxycytidine), in combination with temozolomide, radiation or even oncolytic viruses, have been shown to hold beneficial effects in in vitro and in vivo glioma models [43–45]. However, despite these pre-clinical tests and a few clinical trials (see clinicaltrials.gov), DNA demethylating agents have failed to establish themselves as part of the treatment protocols for high-grade gliomas. Therefore, more targeted strategies in terms of both the molecular and the cellular targets should be considered. Our findings that glioma cells induce a significant decrease in DNMT3A expression in microglia, potentially conferring anti-tumoral functions, underscore the need to investigate whether microglial DNMT3A can be selectively targeted to treat glioma. Interestingly, our data indicate that TGF-β1 signaling is also modulated in our model, suggesting that glioma-induced microglial reprogramming may involve not only DNMT3A-dependent epigenetic changes but also alterations in pathways such as TGF-β1; future studies should explore the mechanistic interplay between these processes.

RNA-based antisense technology is reported to be an efficient approach for selectively reducing DNMT protein expression [46, 47], and this strategy has even attracted interest within the glioma research field [48]. In addition, microglia have been considered an interesting cellular target for glioma treatment; however, recent efforts have focused on repressing their tumor-supporting functions or even achieving their depletion (for review see [49]). Instead, we propose to capitalize on the in-built anti-tumoral properties of microglia, which we have now discovered can be stimulated by the cancer cells themselves.

Our investigations reveal that i) the expression of DNMT3A can be selectively and robustly reduced in microglia using RNA-based antisense approaches, transiently using small interfering RNA and even in a stable manner using small hairpin RNA, ii) that reduced DNMT3A expression is associated with a microglial phenotype exhibiting anti-tumoral characteristics, and finally iii) that targeting microglial *Dnmt3a* using ASOs impacted negatively on the growth of GB tumors in an animal model of the disease. Thus, by leveraging a glioma-induced microglial signaling pathway that drives the acquisition of a transient phenotype characterized by inflammatory and immune response features, we can target and treat the neoplastic cells. Furthermore, the modulation of microglia DNMT3A expression could provide an option to override the immune inhibitory and tumor-supportive functions exerted by these cells in the context of the glioma tumor microenvironment and should therefore be further considered as a therapeutic strategy.

## Methods

### Cell culture

BV-2 (referred as BV2) mouse microglia cell line (RRID: CVCL_0182; gift of G. Brown, University of Cambridge) [11, 19, 50], HMC3 human microglia cell line (RRID: CVCL_II76; also known as CHME3, obtained from originator M. Tardieu, Paris-Sub University) [51], GL261 mouse GB, IDH1 wildtype, cell line expressing the enhanced green fluorescent protein (GFP) (referred as GFP-GL261) (RRID: CVCL_Y003; gift of R. Glass, Max Delbrück Center) [11, 26] and C6 rat glioma, IDH1 wildtype, cell line (RRID: CVCL_0194; gift of O. Hermanson, Karolinska Institutet) and C8-D1A mouse astrocytic cell line (RRID: CRL-2541; purchased from ATCC, CRL-2541™) were cultivated in DMEM + glutamax medium (Gibco) supplemented with 10% FBS (fetal bovine serum) and 1% P/S (Penicillin/Streptomycin). MN9D mouse dopaminergic neuronal cell line (RRID: CVCL_M067; obtained from the originator Alfred Heller, University of Chicago) was cultivated in DMEM/F12 glutamax medium (Gibco) supplemented with 10% FBS and 1% P/S. U-87MG ATCC (referred to as U87-MG) human GB, IDH1 wildtype, cell line (RRID: CVCL_0022; purchased from ATCC, HTB-14™) was cultivated in MEM medium supplemented with 10% FBS and 1% P/S. All cell lines were grown in an incubator at 37°C and 5% CO2 and regularly tested with Venor GeM mycoplasma detection kit (Minerva Biolabs).

For segregated coculture experiments, microglial cells were first seeded in 5% FBS medium on coverslips in 12-well plates, while glioblastoma neuronal and astrocytic cells were seeded in 94 mm diameter Petri dishes. Thereafter, 24 h after seeding, the cocultures were initiated by placing the coverslips with microglial cells into cell strainers in the petri dishes containing the glioblastoma cells. Microglial cells were then harvested at given time-points.

### Glial cell extraction and following microglia isolation

Glial cells were isolated from the brains of male C57BL/6J mice (Charles River, Sulzfeld, Germany, stock #000664) on postnatal day 14. All the experimental procedures were carried out according to the European and Swedish animal welfare regulations approved by the northern Stockholm ethical committee (application no. 13676-2020) (*n*  =  10 per preparation). Animals were deeply anesthetized with sodium pentobarbital (ABCUR AB, Sweden), and transcardially perfused with ice-cold 1 × phosphate-buffered saline without Ca2+ and Mg2+ (PBS; pH 7.4; GIBCO/Life Technologies #10010056). Brains were collected, the cerebella and olfactory bulbs were removed, and cerebra were placed into 50 mL tubes containing 1 × PBS and kept on ice. Briefly, dissected cerebra were transferred to Petri dishes and finely minced using scalpels. The tissue was then enzymatically digested in 2.5 mL of an enzyme solution consisting of 0.01% papain (0.1%; Roche #000000010108014001), 0.1% dispase II (Sigma-Aldrich #D4693), 0.05% DNase I (Roche # 000000010104159001), and 12.4 mM MgSO₄ (Sigma-Aldrich #M7506) made in 1× Hank’s-buffered salt solution (HBSS) without Ca2+ and Mg2+ (GIBCO/Life Technologies #14175095). Samples were incubated at 37 °C for 10 min, after which the enzymatic activities were stopped with 20% cold heat-inactivated fetal bovine serum (FBS; GIBCO/Life Technologies #10500064). Tissue homogenization was achieved by gentle trituration using a pipette until a uniform suspension was obtained. Cell suspensions were filtered through a 70 μm strainer, diluted with 20% FBS in HBSS, and centrifuged at 500 × g for 5 min at 4 °C. After washing with 1× HBSS, cells were resuspended in 20% percoll solution (percoll plus, GE Healthcare, #GE17-0891-02); 10× phenol red HBSS (Gibco/Life Technologies #14060040) and 1× HBSS, and overlaid with an equal volume of 1× HBSS, and overlaid carefully with 1× HBSS, and centrifuged at 500 × g for 20 min at 4 °C without brake to remove the myelin. Following removal of the supernatant, cells were washed in 1× HBSS and centrifuged again at 500 × g for 5 min at 4 °C. The final pellet was resuspended in complete culture medium consisting of DMEM/F12 with Glutamax culture medium (Gibco/Life Technologies #31331028) and 10% heat-inactivated FBS, supplemented with 10 ng/mL recombinant mouse M-CSF (R&D Systems 416-ML-010). Cells were plated in a T75 flask and grown at 37 °C in 5% CO2. The culture media were replaced every 2–3 days.

### Gene silencing by transfection of small interfering RNAs pools

For transient gene expression silencing by siRNAs, non-targeting control, *Dnmt1*, *Dnmt3a* and *Dnmt3b* ON-TARGET plus SMARTpools siRNAs, whose sequences can be found in the Supplementary Table 1, were obtained from Dharmacon. Transfection of BV2 cells was carried out with Lipofectamine 3000 (Invitrogen).

Primary microglia were transfected with siRNA using the Glial-Mag transfection reagent and magnetic plate system (OZ Biosciences) according to the manufacturer’s protocol with minor modifications. The procedure was performed in a 24-well low evaporation plate, with reagent volumes adjusted accordingly. For each well, 0,6 μL of siRNA (20 μM stock concentration) was diluted in 90 μL DMEM/F12 without serum or antibiotics and 0,5 μL of Glial-Mag reagent was mixed gently. The mixture was incubated at room temperature for 20 min to allow complex formation. Meanwhile, the culture medium in each well was replaced with 330 μL fresh DMEM/F12 without serum or antibiotics. Following incubation, the siRNA–Glial-Mag complex was added dropwise to each well along with 4 μL of Glial-Boost (100×), as recommended in the kit protocol. The culture plates were then placed on the magnetic plate inside a humidified incubator at 37 °C with 5% CO_2_ for 30 min to facilitate magnetic-assisted transfection. After removing the magnetic plate, cells were incubated for an additional 3 h under the same conditions. Subsequently, the medium was replaced with complete culture medium (DMEM/F12 supplemented with 10% FBS and 1% P/S), and cells were allowed to recover for 24 h prior to the transwell migration assay.

### Dnmt3a gene silencing by short hairpin RNA lentiviral infection

For stable gene expression silencing of *Dnmt3a* by shRNA, MISSION® shRNA for *Dnmt3a* in pLKO-PURO vector in lentiviral particles, and an empty pLKO-PURO vector used as a control, were purchased from Sigma Aldrich. Two clones (Clone IDs: TRCN0000039034 with target sequence: CCAGATGTTCTTTGCCAATAA, and TRCN0000039035 with target sequence: GCAGACCAACATCGAATCCAT) were tested for lentiviral infection at different Multiplicity of Infection (MOI 1, MOI 2 and MOI 5) of the BV2 cells overnight in the presence of polybrene (Sigma-Aldrich). The MOI of 2 was selected as the best ratio of infection. Two days post-infection, cells that incorporated the shRNA were selected using fresh medium containing 5 µg/ml of Puromycin (Sigma-Aldrich) and shRNA knockdown efficiency was monitored by immunoblot analysis (as described below) after the first 3 cell passages and at regular intervals when running experimental work.

### Cell death

The cell apoptosis was detected using an Annexin V-FITC/PI apoptosis kit (Fisher #10267392) according to the manufacturer´s protocol. At the indicated time and conditions, the media and cells digested with TrypLE Express Enzyme (1X) (Fisher #10718463) were collected and washed with PBS. In the segregated coculture experiments, tumor cells and microglia cells were pooled together, and CD11b staining (Brilliant Violet 421, Biolegend #101236) was performed to distinguish both populations. Subsequently, cells were incubated in 100 μL Annexin binding buffer with Annexin-V- FITC (5 μL) and Propidium Iodide (0,5 μL) for 15 min at room temperature. The apoptosis rate was analysed using a FCM flow cytometer (BDFACS Canto II, Biomedicum Flow Cytometry Core Facility, Karolinska Institute) according to the instructions.

### Immunoblotting

Total protein extracts were made directly in Laemmli buffer by scraping the cells. For immunoblot analysis, protein extracts were resolved on 8% SDS–polyacrylamide gel electrophoresis and then blotted onto 0.45 µm pore-size nitrocellulose membranes. Membranes were blocked with 0.1% Tween/5% milk in PBS and incubated with the indicated primary antibodies, overnight at 4 °C, followed by incubation with the appropriate horseradish peroxidase secondary antibody (Pierce, 1:10,000) for 1 h at room temperature 20–25 °C. An immunoblot with anti-β-actin antibodies was used for standardization of protein loading. Details about antibodies used in this study can be found in Supplementary Table 2. Bands were visualized by enhanced chemiluminescence (ECL-Plus, Pierce) following the manufacturer’s protocol. Alternatively, the membranes were incubated with the appropriate secondary antibody, RDye® 680RD Goat anti-Rabbit or RDye® 800CW Goat anti-Mouse IgG (1:5000; LI-COR Bioscience, Lincoln, NE, USA) for 1 h at room temperature and visualized using an Odyssey CLx infrared imaging system (LI-COR Bioscience). All targeted proteins of interest were normalized to the selected housekeeping gene, the intensity of the bands was verified within the same linear range, and quantification was performed using the ImageJ software. The uncropped immunoblots can be found in Supplementary Data S4.

### DNA extraction

DNA extraction was performed using the QIAamp DNA Mini Kit (Qiagen). DNA concentration was quantified using a NanoDrop® spectrophotometer (Thermo Fisher Scientific).

### DNA methylation analysis

Whole-genome DNA methylation analysis was performed using the Illumina® Infinium HumanMethylation450 BeadChip array. Experiments were performed according to protocol at the Bioinformatics and Expression Analysis (BEA) Core facility at Novum, Karolinska Institutet.

### Dot blot

500 µg of DNA in 0.1 M NaOH (or positive 5mC DNA used as control, Diagenode) were denaturated 5 min at 99˚C. The samples were then neutralized with 0.1 volume of 6.6 M ammonium acetate and spotted on a Hybond-N+ membrane (Amersham). To assess DNA loading, the membrane was stained with methylene blue (0.04% in 0.5 M sodium acetate, pH 5.2) for 5 minutes at room temperature, followed by rinsing with distilled water. Images were acquired prior to immunodetection. The membrane was air-dried before cross-linking for 2 h at 80 °C. The membrane was then blocked in 10% milk, 1% BSA diluted in PBS-tween (0.1%) for 1 h at room temperature before the overnight incubation at 4 °C with the primary antibody directed against 5-methylcytosine (Diagenode) diluted 1/250 in blocking solution. After three washes in PBS-tween, the appropriate horseradish peroxidase secondary antibody (Pierce, 1:10,000) was incubated for 1 h at room temperature. Spots were visualized by enhanced chemiluminescence (ECL-Plus, Pierce) following the manufacturer’s protocol. Densitometry was done using the ImageJ software.

### RNA isolation, cDNA synthesis, and qPCR

Total RNA was extracted using the RNeasy Mini Kit (Qiagen). RNA concentrations were quantified using a NanoDrop® spectrophotometer (Thermo Fisher Scientific). BV2 microglia cDNA was synthesized from 1 µg RNA using Oligo dT, dNTPs, and Superscript III Reverse Transcriptase (Invitrogen). Primary microglia cDNA was synthesized from 100 ng RNA using Oligo dT, dNTPs, and Superscript IV Reverse Transcriptase (Invitrogen). qPCR was run on StepOne plus (Applied Biosystems) using the SYBR™ Green master mix (Life Technologies) and primers listed in Supplementary Table 3. *Actb* gene expression in each sample was used for normalization. Results were calculated using the Δ*C*_*t*_ method and represented as a fold over control (microglia cells from monoculture condition).

### RNA sequencing

Total RNA was subjected to quality control with Agilent Tapestation according to the manufacturer’s instructions. 200 ng of Total RNA was subjected to Illumina sequencing, and libraries were prepared with the Illumina TruSeq Stranded mRNA kit, which includes cDNA synthesis, ligation of adapters and amplification of indexed libraries. The yield and quality of the amplified libraries were analysed using Qubit by Thermo Fisher and the Agilent Tapestation. The indexed cDNA libraries were normalized and combined, and the pools were sequenced on the Illumina HiSeq 2000, generating 50 bp single-end reads. Basecalling and demultiplexing were performed using BCL2 software with default settings, generating Fastq files for further downstream mapping and analysis.

### Transcriptome data computational analysis

In vivo *glioma-induced microglia*—A publicly available dataset comparing glioblastoma-associated microglia at 7 days versus 28 days in vivo (GSE246154) was used as the source dataset; genes were selected based on FDR < 0.05 and Fold Change ≥ 1.5. Volcano plot was generated using the online bioinformatics tool VolcaNoseR (https://huygens.science.uva.nl/). The dataset was further analyzed in comparison to the microglial sensome and TGF-β-related gene sets using the Morpheus online tool (https://software.broadinstitute.org/morpheus).

In vitro *glioma-induced microglia*—DEGs between glioma cocultured and monocultured BV2 microglia were analyzed using Volcano plots depicting the up- and down-regulated genes using VolcanoNoseR. All DEGs with a statistically significant *p*-value (*p* < 0.05), regardless of Fold Change, were included for further analysis (Supplementary Data S1). Gene ontology (GO) enrichment for Biological Processes (BP) was performed separately for up- and down-regulated genes across different comparison groups and time points, using the bioinformatics tool Metascape (https://metascape.org) [52]. The top 10 up- and down-regulated GO BP terms are displayed in the main figures; a list of all terms is available in Supplementary Data S2. KEGG pathway analysis for both up- and down-regulated genes was also conducted via Metascape (Supplementary Data S3), and the results were visualized using the SRplot online bioinformatics tool (https://www.bioinformatics.com.cn/srplot) [53]. The in vitro datasets were compared against the microglial sensome and TGF-β gene sets using the Morpheus online tool.

### Chromatin immunoprecipitation (ChIP) and ChIP sequencing

DNMT3A ChIP experiments were done using the HighCell# ChIP kit from Diagenode (kch-mahigh-G48) according to the manufacturer´s instructions. Briefly, after cell cross-linking in 1% formaldehyde and cell lysis, chromatin shearing was done with a Bioruptor® Pico sonicator (Diagenode). Then, each chromatin immunoprecipitation was done using 6.3 μg of antibody. Purified DNA and 1% input were then analyzed by qPCR (primers listed in supplementary Table 2). Data interpretation from qPCR was done by calculating the percentage of input and then normalizing to the control condition.

DNMT3A ChIP-seq experiments were done using the iDeal ChIP-seq kit from Diagenode according to the manufacturer´s instructions. Purified DNA was sent for library preparation and sequencing at the Bioinformatics and Expression Analysis core facility (BEA, Novum, Karolinska Institute).

ChIP DNA was subjected to quality control with Agilent Tapestation according to the manufacturer’s instructions. To construct libraries suitable for Illumina sequencing, the NEB Ultra DNA kit was used. 10 ng of chipped DNA was used as input. The protocol includes ligation of adapters and amplification of indexed libraries and purification with AMpure magnetic beads. The yield and quality of the amplified libraries were analyzed using Qubit by Thermo Fisher and the Agilent Tapestation. The indexed DNA libraries were normalized and combined, and the pools were sequenced on the Illumina HiSeq 2000 for a 50-cycle sequencing run, generating 50 bp single-end reads. Basecalling and demultiplexing were performed using CASAVA software with default settings, generating Fastq files for further downstream mapping and analysis.

### Transwell migration assay

Eight μm-pore-width transparent PET membrane inserts (Transwell, Corning) were used to measure cell migration capability using a transwell system. For the C6 glioma cells migration assay, C6 cells were seeded on top of the insert, and BV2 microglia were seeded in the lower compartment. For the BV2 microglia migration assay, BV2 cells were seeded in the insert in 5% FBS medium, and 10% FBS medium was placed in the bottom as an attractant. Once the experiment was finalized (after 24 h migration for C6 cells and 4 h migration for BV2 cells), the membranes from the inserts were washed with PBS and carefully cut out with a blade. The membranes were mounted with ProLong Gold antifade reagent with DAPI (Life Technologies), and the nuclei of the migrated cells were counted under fluorescent microscopy.

### Phagocytosis assay

pHrodo™ Green Zymozan BioParticles™ Conjugate for Phagocytosis kit (Invitrogen, P35365) was used to assess microglial phagocytosis capability following the manufacturer’s protocol. Cells treated with Cytochalasin D (Calbiochem, #250255), a phagocytosis inhibitor, were used as a control.

### Syngeneic transplant glioma mouse model

C57/BL6/J mice (Charles River) were housed in a 12 h light/12 h dark cycle with access to food and water ad libitum.

*Intrastriatal GL261 GB cells and BV2 microglia injections*—Postnatal day 16–17 male pups were anesthetized with isoflurane (5% for induction and 1.5% for maintenance). An incision was made on the scalp, and the skin flaps were retracted to expose the skull. Animals received an intrastriatal injection of either GL261 GB cells alone (35,000 cells) or a mix of GL261 GB cells and BV2 sh*Dnmt3a* microglia or BV2 shControl microglia (35000 + 15000 cells respectively) resuspended in 1 μl of the culture medium in the left hemisphere using the following coordinates relative to bregma anterior/posterior: +0.7 mm, lateral: ± 2.5 mm, ventral: −3 mm, using a 5 μl ILS microsyringe. The injection was performed over 1 min and the syringe remained in the injection site for 5 min to reduce backflow and was slowly retracted over 1 min thereafter. The skin was sutured, and animals were allowed to recover before they were returned to their dams. Animals were sacrificed 2 weeks after transplantation. The experiment was performed on 4 animals per condition and repeated twice (*n* = 8 animals per condition final).

*Antisense oligonucleotide and cortical GL261 GB cells injections*—ASOs were designed as 5-10-5 MOE gapmers, with mixed phosphorothiate/phosphodiester backbone, to target mouse *Dnmt3a* or no target (Control ASO). ASO sequences were as follows: Control ASO: CCTATAGGACTATCCAGGAA; mouse-specific *Dnmt3a* ASOs: TTTCCATATTTTTATCCATA (ASO-1), ACCTACATTTTAGCACAGCT (ASO-2). Six-week-old mice were anesthetized with isoflurane (5% for induction and 2% for maintenance) using a mouse mask coupled to a stereotaxic apparatus. Mice were also injected subcutaneously (s.c.) with 5 mg/kg Rimadyl (Carprofen) and 0.1 mg/kg buprenorphine (Temgesic) 15 min prior to surgery for systemic anesthesia and analgesia. The fur on top of the head was removed using a shaver, and the skin was disinfected with ethanol. A 2 mm incision was then made on the scalp following lidocaine administration (4 mg/kg). ASOs (*Dnmt3a*-ASO-2, or ASO-Control) or PBS were injected intracerebroventricularly (icv) using the following stereotaxic coordinates: 0.3 mm anterior to bregma (AP), 1.0 mm lateral to bregma (ML), and 3 mm deep (measured from when the orifice of the needle has passed the skull) (DV) using a Hamilton 10 μl syringe (#80030 1701 RN) and a (22 s/51/2)S needle (#7758-03 RN) without drilling the skull. The volume (10 μl) and speed (0.1 μl/sec) of the injection were controlled by an automatic microinjection pump. The syringe remained in the injection site for 3 min to reduce backflow and was slowly retracted thereafter. After the surgery, 2–3 stitches (Ethilon monofilament 5.0) were applied to suture the wound. Eye gel drops (Viscotears 2 mg /ml, # 541760) were used to hydrate the eyes throughout the surgery. Mice (*n* = 12/group) were allowed to recover in a separate cage on a heating pad (35.5 °C) and monitored until they were fully awake and active. They were also given analgesics for post-operative pain relief (Carprofen 5 mg/kg) 24 h later.

Two weeks after the first injections, mice were again anesthetized with isoflurane and administered Rimadyl and buprenorphine s.c. as before. A small craniotomy of 2 mm was performed on the skull using a drill at 1 mm anterior to bregma, 1.5 mm lateral from bregma, and 2.5 mm deep (measured from when the orifice of the needle has passed the hole). A suspension of GL261 GB cells in PBS (50,000 GL261 tumor cells in 3 µl) was injected in all animals using a 10 µl Hamilton syringe (#80030 1701 RN) and a (26/30/4)S needle (#7804-03 RN) at a speed of 1.5 µl/ min. The syringe remained in the injection site for 3 min to reduce backflow and was slowly retracted thereafter. The skin wound was sutured, and the animals were allowed to recover in a separate cage on a heating pad and monitored until they were fully awake and active. They were given analgesics for post- operative pain relief (Carprofen 5 mg/kg, s.c., 24 h later). Animals were sacrificed 2 weeks after cell transplantation.

*Brain tissue extraction, fixation, and sectioning*—Animals were deeply anesthetized with an overdose of sodium pentobarbital and transcardially perfused with 0.9% sodium chloride, followed by fixation with 4% paraformaldehyde in 0.1 M phosphate buffer (pH 7.4). Brains were then transferred to 30% sucrose in 0.1 M phosphate buffer and left until they sank. 25-μm-thick horizontal free-floating sections were prepared using a sliding microtome (Leica SM2010R) and stored in cryoprotection solution at 4 °C (25% glycerol, 25% ethylene glycol in 0.1 M phosphate buffer) for further histological analysis.

### Microglia morphology analysis

CellProfiler (version 4.2.8; Broad Institute Inc.) was used for morphological analysis of microglia. The pipeline used AIF1 IHC (10x magnification) as input and consisted of the identification of objects using adaptive threshold detection (Otsu method, 100 pixels adaptive window). The objects detected were then filtered by morphological features such as area (between 50 and 1000 square microns) and maximum Feret diameter (>5 microns). Object area and solidity (defined as the ratio between the area of the convex hull including the entire object of interest and the object’s actual area) were used for further analyses. Solidity ranges between 0 and 1, and low values (due to high convex hull area, but relatively small area of actual object) indicate ramified structures. Input images were obtained from 4 independent experiments (1 image/animal and experiment). The measurements of microglia from all images per experiment were pooled before statistical analyses (one-way ANOVA followed by Dunnett’s post-hoc test for comparisons against controls as the reference group).

### RNAscope in situ hybridization

RNA in situ hybridization was performed for *Dnmt3a* and *P2ry12* mRNAs. After sacrificing the mice, the brains were immediately frozen on dry ice for 5 min and then embedded in cryo-embedding medium (OCT compound, Sakura Finetek). After equilibrating at –20 °C for 2 h in a cryostat (CryoStar NX70, ThermoScientific), the brains were sectioned into 10 μm slices and mounted onto SuperFrost Plus adhesion slide (Epredia). The slices were dried at -20°C for 1 h and then stored in zip-lock bags at –80 °C until needed. The RNAscope Multiplex Fluorescent Reagent Kit (Advanced Cell Diagnostics) for in situ hybridization assay was used to detect the mRNAs of interest. The slides were fixed by immersion into pre-chilled 4% PFA for 1 h at 4 °C. After rinsing the slides twice with 1X PBS to remove the fixative, the sections were dehydrated in 50%, 70%, 100%, and 100% ethanol each for 5 min at room temperature. RNAscope hydrogen peroxide was added to each slice for 10 min at room temperature, and then washed off with distilled water, followed by protease IV for 10 min at room temperature, and then washed with PBS. *Dnmt3a* (GenBank accession number NM_153743.4; target nt region, 3757–4994) and *P2ry12* (GenBank accession number NM_027571.3; target nt region, 739-1854) probes were pre-warmed for 10 min at 40 °C. The probes targeting RNAs contain different 20 ZZ oligonucleotide probes: Dnmt3a-C1 probe, P2ry12-C3 probe. To hybridize with target RNAs, the probes were mixed and added to the slides for 2 h at 40 °C in a HybEZ oven. The signals were then amplified by AMP1, AMP 2, and AMP 3 hybridization for 30 min at 40 °C, followed by HRP-C1 for 15 min at 40 °C and incubation with Opal^TM^ 570 for 30 min at 40 °C for *Dnmt3a* detection. The slides were then blocked with HRP blocker before adding HRP-C3 and Opal^TM^ 690 for detecting *P2ry12*. DAPI was used for 30 s at room temperature before mounting the slides with Prolong Gold Antifade Mountant. Pictures were taken using a Zeiss LSM900-Airy confocal microscope and analysed with OMERO.insight and ImageJ software.

### Immunohistochemistry

The mice brain free-floating sections were washed in PBS and followed by antigen retrieval treatment using Target retrieval Solution (Dako) by incubation at 80 °C for 30 min and let to rest at room temperature for 30 min. After PBS washes, the sections were permeabilized 15 min in PBS-Triton 1% and then blocked for 1 h at room temperature in 3% serum/0.3% PBS-Triton under gentle shaking. The sections were then incubated with the primary antibodies diluted (as described in supplementary Table 1) in blocking solution overnight at 4 °C. The next day, the sections were washed three times in PBS, followed by incubation in secondary antibody diluted in blocking solution for 2 h at room temperature under gentle shaking. The sections were then washed in PBS, incubated in Hoechst 33342 solution (1:1000 in PBS, Molecular Probes/Life Technologies #H3570) for 10 min at room temperature. After a final PBS wash, the sections were mounted onto SuperFrost Plus adhesion slides (Epredia), let them dry before adding Mounting medium (Prolong Gold Antifade Mounting medium, Invitrogen) and covered with a coverslip. Pictures were taken using a Zeiss LSM800-Airy confocal microscope.

For anti-DNMT3A and AIF1/IBA1 staining, the free-floating mice brain sections were washed several times with 1× Tris-buffered saline (TBS) to remove the cryoprotectant solution. For DNMT3A staining, an intermediate antigen retrieval step was required before blocking. To this end, the brain sections were incubated in sodium citrate solution (NaCl, 10 mM, pH 6.0) for 30 min at 80 °C, allowed to recover for 10 min at room temperature, and then rinsed several times in TBS to remove the excess of NaCi.

Then the sections were subsequently incubated in a blocking solution containing 5% donkey serum (Jackson ImmunoResearch Laboratories, West Grove, PA) and 0.1% Triton X-100, for 1 h at room temperature. Incubation with the primary antibodies ensued for 48 h at 4 °C. The sections were then rinsed with TBS and incubated with the appropriate secondary antibodies for 1 h at room temperature. Hoechst 33342 was added for nuclear counterstaining. Finally, the sections were mounted onto microscope slides using ProLong Gold anti-fade reagent (Molecular Probes/Life Technologies) as mounting media, coverslipped, and allowed to dry at room temperature. Image acquisition was carried out using a Zeiss LSM700 confocal laser scanning microscope equipped with ZEN Zeiss software.

### Tumor volume calculation

Assessment of tumor size and microglial occupancy outcome was blindly analyzed by an experimenter independent from the one who performed animal surgeries. Using a preestablished inclusion criterion, animals presenting a tumor size below 1 mm3 were excluded from the analysis. Volumes in mm^3^ were calculated in coronal sections using the Zeiss software from the GFP-positive and Iba1-positive areas according to the Cavalieri principle using the following algorithm: *V* = Σ*A* × *P* × *T*, where *V* = total volume, Σ*A* = the sum of area measurements, *P* = the inverse of the sampling fraction, and *T* = the section thickness. IBA1 intensity (Arbitrary Unit) using the Zeiss software within the calculated tumor volume was used as a proxy for microglia occupancy within each tumor.

### Statistical analyses and reproducibility

All statistical analyses were conducted using Prism 8/10 (GraphPad Software). Results were tested for statistical significance using one-way ANOVA followed by Dunnett’s post-hoc test for multiple comparisons. If two conditions were to be compared, a two-tailed paired or unpaired Student’s *t* test was used. Results are presented as the mean ± s.e.m. Differences were considered statistically significant if *P* values were less than 0.05. (*P < 0.05, **P < 0.01, and ***P < 0.001). The number of biological replicates is described in the relevant figure legends.

All in vivo experimental studies are guided by the 3 R principle, EU Directive 2010/63/EU. The investigators were blinded to the conditions of the experiments during data collection and quantification. No statistical methods were used to predetermine sample sizes, but these are consistent with previous publications [10, 11, 19, 32]. Animals were randomly assigned to the different experimental groups. For morphological analyses, sample IDs were randomized using an Excel-generated randomized numerical ID.

## Supplementary information

Supplementary_Materials

Data S1

Data S2

Data S3

Data S4

## Data Availability

The transcriptome dataset comparing glioblastoma-associated immune cells including microglia at 7 days versus 28 days in vivo is available at the Gene Expression Omnibus with accession number: G. Raw data for the transcriptome dataset comparing in vitro glioma-stimulated microglia versus microglia alone are available in the Supplementary Data S1 as well as at the Gene Expression Omnibus with accession number: G. Raw data for the DNMT3A ChIP-seq dataset comparing in vitro glioma-stimulated microglia versus microglia alone are available s at the Gene Expression Omnibus with accession number: G. All other data are available from the corresponding author upon reasonable request.
