# Supplementary material for: Glioma-induced DNMT3A reduction in microglia promotes an anti-tumoral phenotype
Source: Cell Death Differ. 2026 Mar 18;33(10):1960–78. doi: 10.1038/s41418-026-01712-x (PMC13631343; doi:10.1038/s41418-026-01712-x)
Supplement: Supplementary file 1 [file 41418_2026_1712_MOESM1_ESM.docx]

Supplementary Materials for

**Glioma-induced DNMT3A reduction in microglia promotes an anti-tumoral phenotype.**

Mathilde Cheray, Mercedes Posada-Pérez, Adamantia Fragkopoulou, Carlos F.D. Rodrigues, Adriana-Natalia Murgoci, Ahmed M. Osman, Guillermo Vázquez-Cabrera, Martin Škandík, Christine Hong, Pinelopi Engskog-Vlachos, Shigeaki Kanatani, Yue Li, Stefan Spulber, Lara Friess, Theodora Sylaidi, Marie-Kim St Pierre, Lena-Maria Carlson, Anastasius Damdimopoulos, Per Uhlén, Fredrik Kamme, Klas Blomgren, and Bertrand Joseph.

**This PDF file includes:**

Extended Data Figures 1 to 7

Tables S1 to S3

Captions for Data S1 to S4

**Other Supplementary Materials for this manuscript include the following:**

Data S1 Raw data RNA-seq 2h and 4h (RNA-seq.xls)

Data S2 GO BP enrichment analysis (GO BP.xls)

Data S3 KEGG pathways analysis (KEGG.xls)

Data S4 Uncropped immunoblots (Immunoblots.pdf)

**Extended Data Figure 1 | Decreased DNMT3A expression, and induction of *Il1β* mRNA expression are observed in a BV2 microglia exposed to GL261 glioblastoma cells.**

**a**,**b**, Immunoblot analysis (**a**), with quantifications (**b**), of DNMT3 and β-actin (ACTB) expression levels in murine BV2 microglia in monoculture or in coculture with murine GL261 GB cells at the indicated time-points. **c**, RTqPCR analysis of *Dnmt1*, *Dnmt3a*, and *Dnmt3b* mRNA expression levels in BV2 microglia exposed to coculture with GL261 GB cells for 2h, 4h, and 6h. **d**, RTqPCR analysis of *IL1b,* *IL6,* and *Mmp14* mRNA expression levels in BV2 microglia exposed to coculture with GL261 GB cells for 2h, 4h, and 6h. **P* < 0.05 (pair comparisons, two-tailed Student's *t* test) (a, and b, n=3, c, and d, n=7; Data are shown as mean ± sem).

**Extended Data Figure 2 | Decreased DNMT3A expression, and induction of *IL1β* mRNA expression are observed in a HMC3 microglia exposed to U87-MG glioblastoma cells.**

**a**,**b**, Immunoblot analysis (**a**), with quantifications (**b**), of DNMT3 and β-actin (ACTB) expression levels in HMC3 microglia in monoculture or in coculture with U87-MG GB cells at the indicated time-points. **c**, RTqPCR analysis of *DNMT1*, *DNMT3A*, and *DNMT3B* mRNA expression levels in human HMC3 microglia exposed to coculture with U87-MG glioblastoma cells for 2h, 4h, and 6h. **d**, RTqPCR analysis of *IL1b* and *IL6* mRNA expression levels in human HMC3 microglia exposed to coculture with U87-MG GB cells for 2h, 4h, and 6h. **P* < 0.05, and ***P* < 0.01 (pair comparisons, two-tailed Student's *t* test) (a and b, n=5; c and d, n=3; Data are shown as mean ± sem).

**Extended Data Figure 3 | Stimulation of BV2 microglia with LPS or IL4 treatments do not mimic the effects observed upon exposure to C6 glioma cells.**

**a**,**b**, Immunoblot analysis (**a**) of DNMT3, arginase-1 (ARG1), nitric oxide synthase 2 (NOS2), and β-actin (ACTB) expression levels in BV2 microglia treated with LPS or IL4 for 2h, 4h, 6h, and 24 h with quantifications for DNMT3A (**b**). **c**,**d**, RTqPCR analysis of mRNA expression levels in BV2 microglia treated with LPS (**c**) or IL4 (**d**) for 2h, 4h, 6h, and 24 h of *Dnmt1*, *Dnmt3a*, and *Dnmt3b*. **P* < 0.05, ***P* < 0.01, and ****P* < 0.001 (pair comparisons, two-tailed Student's *t* test) (a-d, n=3; Data are shown as mean ± sem).

**Extended Data Figure 4 | Absence of induction of *Il1β* or *Il6* mRNA expression in a BV2 microglia exposed to NM9D dopaminergic neuron or C8-D1A** **astrocytes.**

**a-d**, RTqPCR analysis of *IL1b* (**a**,**c**) and *IL6* (**b**,**d**) mRNA expression levels in BV2 microglia exposed to coculture with NM9D dopaminergic neuron (**a**,**b**) or C8-D1A astrocytes (**c**,**d**) for 2h, 4h, and 6h. **P* < 0.05, ***P* < 0.01, and ****P* < 0.001 (pair comparisons, two-tailed Student's *t* test) (n=4; Data are shown as mean ± sem).

**Extended Data Figure 5 | Co-culture of BV2 microglia with C6 glioma cells or GL261 GB cells, or *Dnmt3a* silencing in BV2 microglia do not affect their survival.**

**a**,**b**, flow cytometric analysis of cell death using Annexin V/Propidium Iodide staining in C6 glioma cells and BV2 microglia (**a**) or GL261 GB cells and BV2 microglia (**b**) cocultured for 24 hours. **c**, flow cytometric analysis of cell death using Annexin V/Propidium Iodide staining in

BV2 microglia transfected with a pool of siRNAs targeting *Dnmt3a* expression (siRNA-*Dnmt3a*), as compared to cells transfected with a pool of non-targeting siRNAs control (siRNA-Control/Ctrl, negative control). (pair comparisons, two-tailed Student's *t* test) (n=4, Data, set to 100%, are shown as mean ± sem)

**Extended Data Figure 6 | Repression of *Dnmt1* or *Dnmt3b* expression in BV2 microglia do not mimic the effect observed upon *Dnmt3a* silencing.**

**a,** RTqPCR analysis of *Dnmt1*, *Dnmt3a*, *Dnmt3b* mRNA expression levels in BV2 microglia transfected with a pool of siRNAs targeting *Dnmt1* expression (siRNA-*Dnmt1*), or *Dnmt3b* expression (siRNA-*Dnmt3b*), as compared to cells transfected with siRNA-Control/Ctrl, negative control, set as 1. **b**,**c**, Immunoblot analysis of DNMT1 (**b**), or DNMT3B (**c**) together with β-actin (ACTB) expression levels in mock transfected (-) BV2 microglia, or cells transfected with siRNA-Ctrl, siRNA-*Dnmt1* (**b**) or siRNA-*Dnmt3b* (**c**). **d**, Analysis of cell migration capability of mock transfected (-) BV2 microglia, or cells transfected with siRNA-Ctrl, siRNA-*Dnmt1,* or siRNA-*Dnmt3b.* **e**, Analysis of C6 glioma cell migration capability toward mock transfected (-) BV2 microglia, or cells transfected with siRNA-Ctrl, siRNA-*Dnmt1,* or siRNA-*Dnmt3b.* **P* < 0.05, ***P* < 0.01, and ****P* < 0.001 (pair comparisons, two-tailed Student's *t* test) (a-e, n=3; Data are shown as mean ± sem).

**Extended Data Figure 7 | *In vivo*, antisense oligonucleotides targeting *Dnmt3a* promote microglial galectin3 expression.**

**a**, Confocal immunofluorescence imaging showing galectin-3 (LGALS3, green), and IBA1-expressing microglia (red) in brain tissues of mice injected with ASO-Ct or ASO targeting DNMT3A (ASO-1 and ASO-2). Hoechst (blue) as nuclear counterstain. (n=3, Scale bars 1000µm). Zooms in merge images corresponding to the white doted boxes are presented on the right-hand side. **b**, Confocal immunofluorescence imaging of tumors formed 2 weeks post injection of GFP-GL261 GB cells (green) in mice previously treated with PBS, or ASO-Ct (as illustrated in c) together with an immunostaining for the microglial marker IBA1 (red) and Hoechst used as nuclear counterstain (blue). **c**,**d**, Quantification of 2 weeks old GL261 GB tumor volume (**c**) and microglia occupancy (**d**) in mice injected with PBS or ASO-Ct, 2 weeks prior injection of GFP-GL261 GB cells. (n=8/9 mice per group; pair comparisons, two-tailed Student's *t* test; Data are presented as box plot with data point for individual mice depicted as circle)(Scale bars 100µm).

Table S1 | ON-TARGETplus SMART pool small interfering RNAs used in this study.

| **ON-TARGET plus SMARTpools siRNAs** | **Companies** |
| --- | --- |
|  |  |
| ***Dnmt1*** (mouse, DNMT1 NM_010066) | Dharmacon (L-056796) |
| GGUAGAGAGUUACGACGAA |  |
| AAGCAAUUCAUGACGAGAA |  |
| GGUCGUGAGUGUUCGGGAA |  |
| GCUGGGAGAUGGCGUCAUA |  |
|  |  |
| ***Dnmt3a*** (mouse, DNMT3A NM_001271753) | Dharmacon (L-065433) |
| CCGUGAUGAUUGACGCCAA |  |
| GGUCCUAGGAGGCGAACUU |  |
| CCGCAAAGCCAUCUACGAA |  |
| CCAAAGCAGCCGACGAUGA |  |
|  |  |
| ***Dnmt3b*** (mouse, DNMT3B NM_001271745) | Dharmacon (L-044164) |
| GAGGAGUGCAUUAUCGUUA |  |
| UCAGGAUGAUAAAGAGUUU |  |
| GCAAUGAUCUCUCUAACGU |  |
| GGAAUGCGCUGGGUACAGU |  |
|  |  |
| **Non-targeting siRNA pool** (si-Control) | Dharmacon (D-001810) |
| UGGUUUACAUGUCGACUAA |  |
| UGGUUUACAUGUUGUGUGA |  |
| UGGUUUACAUGUUUUCUGA |  |
| UGGUUUACAUGUUUUCCUA |  |
|  |  |

Table S2 | Antibodies used in this study

| **Primary Antibodies** | **Company** | **Dilution** |
| --- | --- | --- |
| **5-Methylcytosine** (33D3; Mouse mAb)  **DNMT1** (D63A6; Rabbit mAb)  **DNMT3A** (ChIP grade; rabbit pAb)  **DNMT3A** (H-295; rabbit pAb)  **DNMT3A** (rabbit pAb)  **DNMT3B** (52A1018 ChIP grade; mouse mAb)  **ATCB/β-Actin** (AC40; mouse mAb)  **LGALS3/Galectin-3** (Goat pAb)  **GFP** (Goat pAb)  **AIF1/IBA1** (Rabbit pAb)  **AIF1/IBA1** (Goat pAb)  **ARG1/Arginase 1** (V-20; goat pAb)  **NOS2** (M19; rabbit pAB)  Brillant Violet 421-**CD11b** (M1/70; rat mAB) | Diagenode (C15200081)  Cell Signaling (#5032)  Abcam (ab2850)  Santa Cruz Biotech(sc-20703)  Abcam (ab188470)  Abcam (ab13604)  Sigma Aldrich (A-3853)  R&D Systems (AF1197)  Abcam (ab6673)  Wako (019-19741)  Abcam (5076)  Santa Cruz Biotech(sc-18354)  Santa Cruz Biotech(sc-650)  Biolegend (#101236) | 1/250  1/1000  1/500  1/500  1/500  1/500  1/2000  1/500  1/1000  1/1000  1/500  1/1000  1/1000  FACS |
| **Secondary Antibodies** | **Company** | **Dilution** |
| **RDye® 800CW Goat anti-Mouse**  **RDye® 680RD Goat anti-Rabbit**  **Donkey anti-Goat Alexa fluor 488**  **Donkey anti-Goat Alexa fluor 555**  **Donkey anti-Rabbit Alexa fluor 488**  **Donkey anti-Rabbit Alexa fluor 555**  **Donkey anti-Rabbit Alexa fluor 594** | LI-COR Biosci(926-32210)  LI-COR Biosci (926-68071  Invitrogen (A11055)  Invitrogen (A21432)  Invitrogen (21206)  Invitrogen (A31572)  Invitrogen (A21207) | 1/5000  1/5000  1/200  1/200  1/200  1/200  1/200 |

**Table S3 | Primer sequences**

All sequences are given 5´to 3´

| **cDNA (organism)** | **Forward primer** | **Reverse primer** |
| --- | --- | --- |
| ***Actb*** (mouse) | TTGCTGACAGGATGCAGAAG | TGATCCACATCTGCTGGAAG |
| ***Dnmt1*** (mouse) | GTGAACAGGAAGATGACAAC | CTGGATCCTCCTTTGATTTC |
| ***Dnmt3a*** (mouse) | ACCAGAAGAAGAGAAGAATCC | CAATGATCTCCTTGACCTTAG |
| ***Dnmt3b*** (mouse) | GACTTCATGGAAGAAGTGAC | TATCATCCTGATACTCTGTGC |
| ***Il1β*** (mouse) | GCTGCTTCCAAACCTTTGAC | TTCTCCACAGCCACAATGAG |
| ***Il6*** (mouse) | GGACCAAGACCATCCAATTC | GGCATAACGCACTAGGTTTG |
| ***Mmp2*** *(mouse)* | GAGATCTTCTTCTTCAAGGAC | AATAGACCCAGTACTCATTCC |
| ***Mmp14*** *(mouse)* | TCCGGATAAGTTTGGGACTG | CATTATGCTGCCACTTGAGG |
| ***ACTB*** (human) | GACGACATGGAGAAAATCTG | ATGATCTGGGTCATCTTCTC |
| ***DNMT1*** (human) | CGTAAAGAAGAATTATCCGAGG | GTTTTCTAGACGTCCATTCAC |
| ***DNMT3A*** (human) | GAAGAGAAGAATCCCTACAAAG | CAATAATCTCCTTGACCTTGG |
| ***DNMT3B*** (human) | CTTACCTTACCATCGACCTC | ATCCTGATACTCTGAACTGTC |
| ***IL1β*** (human) | CTAAACAGATGAAGTGCTCC | GGTCATTCTCCTGGAAGG |
| ***IL6*** (human) | GCAGAAAAAGGCAAAGAATC | CTACATTTGCCGAAGAGC |
|  |  |  |
| ***Il1β* ChIP** (mouse) | EpiTect ChIP qPCR Primer (Qiagen) | (NM_008361.3 (+) 01 kb) |
| ***Il6* ChIP** (mouse) | EpiTect ChIP qPCR Primer (Qiagen) | (NM_031168.1 (+) 01 kb) |
| ***Mmp14* ChIP** (mouse) | EpiTect ChIP qPCR Primer (Qiagen) | (NM_008608.2 (+) 01 kb) |
|  |  |  |

**Caption for Data. S1 | Raw data of RNA-seq 2h and 4h for C6 glioma-stimulated BV2 microglia (segregated co-culture) versus BV2 microglia alone (monoculture).**

**Caption for Data. S2 | Gene ontology (GO) enrichment for biological processes (BP) analyses performed separately for up- and down-regulated genes comparing DEGs in C6 glioma-stimulated BV2 microglia (segregated co-culture) versus BV2 microglia alone (monoculture) at 2h and 4h time points.** (The top 10 up- and down-regulated GO BP terms are displayed in Figure 1).

**Caption for Data. S3 | Kyoto Encyclopedia of Genes and Genomes (KEGG) PATHWAY** **analyses performed separately for up- and down-regulated genes comparing DEGs in C6 glioma-stimulated BV2 microglia (segregated co-culture) versus BV2 microglia alone (monoculture) at 2h and 4h time points.**

**Caption for Data. S4 | Uncropped immunoblots.**
